# Integrating isotopic and nutritional niches reveals multiple dimensions of individual diet specialisation in a marine apex predator

**DOI:** 10.1111/1365-2656.13852

**Published:** 2022-12-23

**Authors:** Richard Grainger, Vincent Raoult, Victor M. Peddemors, Gabriel E. Machovsky‐Capuska, Troy F. Gaston, David Raubenheimer

**Affiliations:** ^1^ Charles Perkins Centre The University of Sydney Sydney New South Wales Australia; ^2^ School of Life and Environmental Sciences The University of Sydney Sydney New South Wales Australia; ^3^ School of Environmental and Life Sciences University of Newcastle Ourimbah New South Wales Australia; ^4^ New South Wales Department of Primary Industries, Fisheries Sydney Institute of Marine Science Mosman New South Wales Australia; ^5^ Nutri Lens East Ryde New South Wales Australia

**Keywords:** *Carcharodon carcharias*, individual specialisation, marine predators, multidimensional nutritional niche framework, nutritional ecology, stable isotopes, tooth replacement

## Abstract

1. Dietary specialisations are important determinants of ecological structure, particularly in species with high per‐capita trophic influence like marine apex predators. These species are, however, among the most challenging in which to establish spatiotemporally integrated diets.
2. We introduce a novel integration of stable isotopes with a multidimensional nutritional niche framework that addresses the challenges of establishing spatiotemporally integrated nutritional niches in wild populations, and apply the framework to explore individual diet specialisation in a marine apex predator, the white shark *Carcharodon carcharias*.
3. Sequential tooth files were sampled from juvenile white sharks to establish individual isotopic (δ‐space; δ^13^C, δ^15^N, δ^34^S) niche specialisation. Bayesian mixing models were then used to reveal individual‐level prey (p‐space) specialisation, and further combined with nutritional geometry models to quantify the nutritional (N‐space) dimensions of individual specialisation, and their relationships to prey use.
4. Isotopic and mixing model analyses indicated juvenile white sharks as individual specialists within a broader, generalist, population niche. Individual sharks differed in their consumption of several important mesopredator species, which suggested among‐individual variance in trophic roles in either pelagic or benthic food webs. However, variation in nutrient intakes was small and not consistently correlated with differences in prey use, suggesting white sharks as nutritional specialists and that individuals could use functionally and nutritionally different prey as complementary means to achieve a common nutritional goal.
5. We identify how degrees of individual specialisation can differ between niche spaces (δ‐, p‐ or N‐space), the physiological and ecological implications of this, and argue that integrating nutrition can provide stronger, mechanistic links between diet specialisation and its intrinsic (fitness/performance) and extrinsic (ecological) outcomes. Our time‐integrated framework is adaptable for examining the nutritional consequences and drivers of food use variation at the individual, population or species level.

## Graphical abstract

The authors introduce an integration of stable isotopes and nutritional geometry for examining time‐integrated nutrition in wild populations. Using this framework, they reveal individual variation in prey use, ecological roles, and nutrition in white sharks. This approach can establish the nutritional consequences of diet variation across individuals, populations, or species.

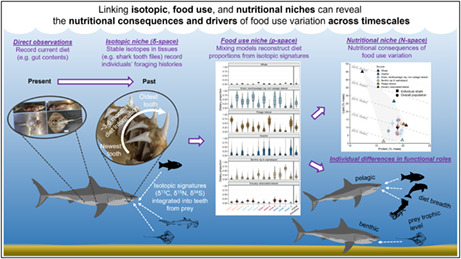

## 1 INTRODUCTION

Trophic interactions are key determinants of ecosystem structure and function and understanding these can help predict the impacts and persistence of organisms across environmental contexts (Machovsky‐Capuska, Senior, et al., 2016b; Rader et al., 2017; Senior et al., 2016; Slatyer et al., 2013). Nonetheless, adequately quantifying diet in free‐ranging animals remains a significant challenge, especially for cryptic species, because direct foraging observations are often unfeasible or spatiotemporally restricted. Stable isotopes (SI) are a widely adopted solution for establishing time‐integrated diets because they assimilate foraging information (food use and/or foraging habitat) over periods defined by rates of consumer tissue isotopic turnover (Ramos & Gonzalez‐Solis, 2012). Carbon and nitrogen SI are most commonly measured, separating primary production sources (δ^13^C) and trophic levels (δ^15^N), although other SI can help distinguish additional foraging attributes (δ^18^O, δ^2^H, habitat use; deHart & Picco, 2015; δ^34^S, pelagic vs benthic feeding; Raoult et al., 2019). “Isotopic niches” (Newsome et al., 2007) are thus widely adopted as proxies for foraging niches (despite caveats; Shipley & Matich, 2020), enabling standardised comparisons across ecological hierarchies, from individuals to communities (Ingram et al., 2018; Jackson et al., 2011). While δ‐space metrics offer useful insights into realised foraging niches, translation to proportional resource use (p‐space) through Bayesian SI mixing models is a requisite step for linking diet specialisation to its ecological outcomes (Newsome et al., 2012; Stock et al., 2018).

Establishing the ecological consequences of dietary specialisation (“extrinsic” outcomes) is usually a focus for isotopic (δ‐ and p‐space) approaches, but implications regarding consumer resource acquisition, physiological performance and fitness (“intrinsic” outcomes) are equally important, albeit underexplored (but see Costa‐Pereira, Toscano, et al., 2019b). Studies across trophic guilds consistently demonstrate how nutrition, and regulation of macronutrient (protein, lipid, carbohydrate) balance, mediates feeding choices (Behmer, 2009; Coogan et al., 2017; Erlenbach et al., 2014; Felton et al., 2016; Hewson‐Hughes et al., 2013; Raubenheimer et al., 2005; Rowe et al., 2018), individual fitness and performance (Jensen et al., 2012; Simpson et al., 2004) and broader ecological phenomena (e.g. migrations, Nie et al., 2015; Raubenheimer et al., 2009; Simpson et al., 2006). To conceptualise these links, Machovsky‐Capuska, Senior, et al. (2016b) introduced a multidimensional nutritional niche framework (MNNF) that models foraging niches across different levels of the dietary hierarchy (e.g. foods, individual meals and overall diets) within a “nutrient‐space” (N‐space) defined using graphical proportions‐based nutritional geometry framework (NGF) models (Raubenheimer, 2011; Raubenheimer et al., 2015).

Although MNNF is widely used in some systems (e.g. primates; Hou et al., 2021; Raubenheimer et al., 2015), field‐based applications have been limited for many species by the difficulty or impossibility of collecting long‐term dietary data, necessitating expedient measures (e.g. stomach contents, regurgitations, scats; Denuncio et al., 2021; Grainger et al., 2020; Machovsky‐Capuska et al., 2018; Senior et al., 2016) that provide only spatiotemporal snapshots of individuals' food and nutrient acquisition. Combining spatiotemporally integrated food biomass assimilation estimates from SI mixing models (Phillips & Koch, 2002) and MNNF could provide a powerful framework for assessing food and nutrient consumption across time scales, but this has not yet been attempted. Moreover, establishing interrelationships in specialisations across different niche spaces (e.g. δ‐, p‐ and N‐space), and their hierarchical partitioning between individuals and species/populations, is necessary for enhancing our understanding of key ecological processes (Carscadden et al., 2020; Takola & Schielzeth, 2022). For instance, examining interplays between variation in food use and nutrient acquisition can reveal animals' nutritional priorities and how physiological requirements are met under ecological constraints (e.g. food availability or competition; Hou et al., 2021; Raubenheimer et al., 2015), which is critical for predicting responses to novel ecological circumstances (Machovsky‐Capuska et al., 2018; Machovsky‐Capuska, Senior, et al., 2016b).

Combining MNNF and SI could enable nutritional assessments at any level of the ecological hierarchy (individuals, populations or species), although quantifying individual‐level nutritional niches is particularly valuable. Specifically, fitness and performance outcomes of diet variation manifest at the individual level (Costa‐Pereira, Toscano, et al., 2019b), and individual diet specialisations, defined where individuals of similar ages/sexes use different subsets of a broader population‐level niche due to phenotypic trait variation, resource availability and competition (Araujo et al., 2011; Bolnick et al., 2002; Svanback & Bolnick, 2007), are increasingly recognised as important determinants of ecosystem dynamics (Bolnick & Ballare, 2020; Ingram et al., 2011). This is particularly important in apex predators because, whilst they generally exert high per‐capita trophic influence on community structure, individual specialisation implies that not all individuals are ecologically equivalent, complicating both our understanding of predators' ecological roles and their management and conservation (Guerra, 2019; Heithaus et al., 2008; Ritchie et al., 2012; Rosenblatt et al., 2015).

Individual specialisation explicitly defines whether individuals' resource use is a subset of that of the overall population, rather than relative to available resources in the environment as per “classical” niche specialisation (Matich et al., 2021; Newsome et al., 2012). Thereby, it is quantitatively formalised using variance partitioning, with total population niche width (TNW) equalling the sum of the between‐individual component (BIC) and within‐individual component (WIC) of variation and individual specialisation increasing as the WIC:TNW ratio decreases (as BIC exceeds WIC; Bolnick et al., 2002; Ingram et al., 2018; Roughgarden, 1972, 1974). Individual variation in resource use can be inferred from SI by comparing tissues with different isotopic turnover rates, or more ideally, using serially accreted, metabolically inert substrates (e.g. hairs, baleen, vertebral and tooth growth bands) that integrate sequential, temporally distinct foraging information (Matich et al., 2021; Newsome et al., 2009; Trueman et al., 2019). While δ‐space individual specialisation has been broadly investigated in marine and terrestrial systems (Costa‐Pereira, Araujo, et al., 2019a; Huckstadt et al., 2012; Matich et al., 2011; Newsome et al., 2009; Noble et al., 2019), expanding similar measures across p‐space (e.g. Newsome et al., 2012) and N‐space is necessary for determining the underlying drivers of individual foraging preferences, their intrinsic (individual) and extrinsic (ecological) outcomes (Machovsky‐Capuska & Raubenheimer, 2020).

Here, we integrate MNNF and SI to explore individual dietary specialisation across multiple niche spaces (δ‐space, p‐space and N‐space) in a marine apex predator, the white shark *Carchardon carcharias*. White sharks are ecologically important yet threatened, cryptic predators (Rigby et al., 2019; Shea et al., 2020). Their diet generally consists of smaller elasmobranchs and teleosts, with the inclusion of larger or higher trophic level prey (e.g. whales, dolphins, sharks) as they transition into subadulthood/adulthood (>3 m total length; Estrada et al., 2006; Grainger et al., 2020; Hussey et al., 2012; Pethybridge et al., 2014; Tamburin et al., 2020). Individual specialisation in white sharks has been inferred previously from whole‐lifetime vertebral SI profiles (annual increments; Kim et al., 2012). However, vertebral profiles are limited for detecting fluctuations in specialisation over shorter timeframes, or through ontogeny (between different years/ages, e.g. Svanback et al., 2015), since they only resolve WIC at interannual or greater timescales (across multiple years). Therefore, we used a novel approach, sampling sequentially formed tooth files, the potential of which for fine‐scale (month increment) individual‐level diet reconstruction in elasmobranchs has been recently highlighted (Shipley et al., 2021; Zeichner et al., 2017), analogous to more widely used systems in other species (e.g. mammalian hair/vibrissae; Newsome et al., 2009). Our specific aims were to (1) link SI and MNNF via mixing models to evaluate individual specialisation at the level of isotopes (δ‐space), food use (p‐space) and nutrient intakes (N‐space), (2) examine potential effects of sex and size on individual specialisation in each niche space and (3) evaluate the relationship between individual specialisation across p‐ and N‐space (i.e. whether individuals are similarly specialised, relative to the population, in both prey use and nutrient intakes). More generally, this illustrates an important application of our broader framework for quantifying time‐integrated nutritional niches in field studies using SI, which could be flexibly adapted across taxa at either the individual, population or species level.

## 2 MATERIALS AND METHODS

### 2.1 Sample collection

**FIGURE 1:**
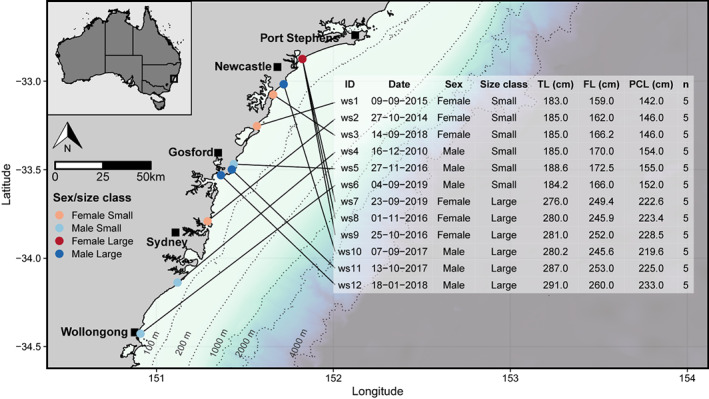
Capture locations, dates, sex, size (total length, TL; fork length, FL; precaudal length, PCL; size class, small and large juveniles) and the number of teeth sampled (n) from white sharks for stable isotope analysis that was included in data analyses. The location of the sampling region in eastern Australia is indicated in the inset map (black square). Shark ID numbers correspond to those used in all other figures. The coastline shapefile and bathymetric data were sourced from the GSHHG Database (Wessel & Smith, 1996; available from https://www.ngdc.noaa.gov/mgg/shorelines/) and GEBCO 2020 15 arc‐second bathymetric grid (GEBCO Compilation Group, 2020; available from https://www.gebco.net/data_and_products/gridded_bathymetry_data/gebco_2020/), respectively.

Teeth were obtained from twelve white sharks caught in the NSW Shark Meshing Program (NSW SMP) operating over the austral summer (September–April) at beaches between Wollongong and Newcastle, Australia (Figure 1). Sharks were caught between 2010 and 2019, but predominantly in September–November from 2014 to 2019 (Figure 1). Multiple males and females from two size classes (small juveniles = ~1.50 m precaudal length (PCL), large juveniles = ~2.25 m PCL) were sampled so that dietary specialisation could be compared across similar individuals (i.e. controlling for size/sex), and between different sexes/size classes (Figure 1). Sampled sharks were of the size range commonly encountered in coastal eastern Australia (Bruce & Bradford, 2012; Spaet et al., 2020). Captured sharks were frozen (−20°C) until necropsy, where sex, PCL, fork length (FL) and total length (TL) were measured, and jaws were excised and refrozen (−20°C) until further processing. Samples of prey species (dolphins, sharks, benthopelagic and benthic rays, pelagic, benthic, reef‐ and estuary‐associated teleosts, cephalopods) consumed by white sharks in eastern Australia based on stomach contents (Grainger et al., 2020) were collected (Table 1). Prey were sampled either through the NSW SMP (bather‐protection nets) or catches from commercial fishers operating off coastal beaches and shelf waters (generally <100 m depth) between Sydney and Port Stephens (Figure 1), an important region within the spatial range of eastern Australian white sharks (Bruce et al., 2019; Spaet et al., 2020). Blubber was collected opportunistically from deceased stranded humpback whales *Megaptera novaeangliae* at Long Reef, Sydney (33.74°S, 151.31°E), Stockton Beach, Newcastle (32.84°S, 151.86°E), and Big Hill Point, near Port Macquarie (31.28°S, 152.97°E), as these may offer an occasional food source for juvenile white sharks (Dicken, 2008; Fallows et al., 2013; Tucker et al., 2019). Prey was stored frozen (−20°C), then partially thawed, measured and weighed, and approximately 1 g of muscle (adjacent to the first dorsal fin for fish and dolphins, central disc musculature for rays, mantle for cephalopods) was excised for isotopic analysis. Blubber was also collected from dolphins since it represents a significant proportion of their total body mass (~21%–26%), in addition to muscle (~26%–37%; Mallette et al., 2016), and a lipid‐rich carbon source for juvenile white sharks (Grainger et al., 2020).

**TABLE 1:** Mean (SD) atomic elemental ratios and isotopic signatures for prey species of white sharks collected in central New South Wales, Australia. Overall average values for source groupings used in mixing models are shown in bold. Values have not been lipid extracted or adjusted for trophic enrichment. For the dolphin source, isotopic signatures and elemental atomic ratios were calculated using weighted averages of δ^13^C, δ^15^N, and elemental concentrations (weight % of C, N and S) in muscle and blubber, weighted by the body mass percentages of each tissue in dolphins (Mallette et al., 2016), since both muscle and blubber are dietary substrates for white sharks. Sulfur was not detected in sufficient quantities in dolphin blubber and thus δ^34^S signatures were from muscle only, and C:S_atomic_ and N:S_atomic_ ratios were undefined for this tissue (weight % S = 0). Abbreviations and source grouping descriptions are in the table footnotes.

Source #	Species (Common name)	Tissue	C:N_atomic_	C:S_atomic_	N:S_atomic_	δ^13^C	δ^15^N	δ^34^S	*n*
(1)	*Megaptera novaeangliae* (Humpback whale)	**B**	**28.9 (21.1)**	**2854.0 (2644.3)**	**89.8 (19.0)**	**−32.6 (1.0)**	**8.3 (2.3)**	**14.4 (3.8)**	**3**
(2)	*Tursiops aduncus* (Bottlenose dolphin)	B	47.4 (27.1)			−22.6 (3.3)	12.9 (3.3)		3
		M	4.7 (1.4)	177.1 (37.5)	38.5 (3.4)	−17.3 (0.8)	15.1 (2.5)	16.5 (2.3)	3
		M, B	9.0 (1.7)	350.1 (38.6)	39.3 (4.0)	−19.8 (1.5)	14.0 (2.4)	16.5 (2.3)	3
	*Delphinus delphis* (Common dolphin)	B	25.0 (17.7)			−20.9 (0.6)	13.6 (0.5)		2
		M	4.2 (0.1)	167.6 (19.4)	40.1 (5.5)	−17.6 (0.2)	13.4 (1.2)	18.5 (0.2)	2
		M, B	7.5 (1.5)	338.8 (5.5)	45.9 (9.8)	−19.2 (0.2)	13.5 (0.4)	18.5 (0.2)	2
	Overall values	**M, B**	**8.4 (1.6)**	**345.6 (28.1)**	**41.9 (6.7)**	**−19.6 (1.1)**	**13.8 (1.8)**	**17.3 (1.9)**	**5**
(3)	*Sphyrna zygaena* (Smooth hammerhead)	M	3.3 (0.0)	142.5 (5.1)	43.1 (1.6)	−17.2 (0.3)	13.8 (1.0)	18.1 (0.9)	6
	*Myliobatis tenuicaudatus* (Southern eagle ray)	M	3.4 (0.2)	131.7 (12.1)	38.6 (5.9)	−15.8 (0.6)	12.4 (0.8)	17.4 (0.3)	8
	*Rhinoptera neglecta* (Cownose ray)	M	3.6 (0.2)	124.7 (5.2)	34.8 (3.2)	−16.1 (0.4)	10.9 (0.4)	17.4 (0.4)	6
	*Pseudocaranx georgianus* (Silver trevally)	M	5.5 (0.9)	196.4 (25.4)	35.5 (1.2)	−18.3 (0.7)	12.5 (0.3)	16.4 (0.2)	8
	*Achoerodus viridis* (Eastern blue groper)	M	3.9 (0.2)	94.5 (9.1)	24.5 (0.9)	−17.8 (0.1)	14.2 (0.4)	17.4 (0.6)	3
	*Kathetostoma leave* (Common stargazer)	M	3.7 (0.1)	107.8 (7.5)	29.4 (1.8)	−16.8 (0.4)	13.5 (0.3)	17.4 (0.6)	6
	*Platycephalus caeruleopunctatus* (Bluespotted flathead)	M	3.8 (0.1)	102.9 (3.4)	27.2 (0.8)	−16.8 (0.3)	13.1 (0.3)	17.8 (0.3)	7
	Overall values	**M**	**3.9 (0.9)**	**133.6 (35.5)**	**34.1 (6.4)**	**−16.9 (1.0)**	**12.8 (1.1)**	**17.4 (0.7)**	**44**
(4)	*Urolophus viridis* (Greenback stingaree)	M	3.1 (0.1)	84.4 (13.0)	27.3 (3.5)	−16.7 (0.5)	13.0 (0.2)	18.3 (1.3)	3
	*Urolophus paucimaculatus* (Sparsely spotted stingaree)	M	2.9 (0.0)	82.8 (4.2)	28.2 (1.5)	−16.8 (0.2)	12.5 (0.3)	18.8 (0.3)	7
	*Hypnos monopterygius* (Coffin ray)	M	2.8 (0.2)	54.0 (8.6)	19.0 (2.3)	−15.6 (0.2)	13.7 (0.6)	20.4 (1.4)	6
	*Sepia rozella* (Rosecone cuttlefish)	M	3.9 (0.0)	54.4 (3.7)	14.1 (1.0)	−16.6 (0.3)	11.4 (0.5)	20.7 (0.9)	8
	*Sepioteuthis australis* (Southern calamari)	M	3.9 (0.0)	97.1 (10.0)	25.1 (2.6)	−16.0 (0.2)	13.4 (0.2)	18.9 (0.5)	8
	Overall values	**M**	**3.4 (0.5)**	**74.0 (19.8)**	**22.1 (6.0)**	**−16.3 (0.5)**	**12.7 (1.0)**	**19.5 (1.2)**	**32**
(5)	*Arripis trutta* (Eastern Australian salmon)	**M**	**3.6 (0.1)**	**135.1 (5.1)**	**37.7 (1.1)**	**−16.8 (0.3)**	**14.3 (0.3)**	**17.6 (0.4)**	**6**
(6)	*Mugil cephalus* (Sea mullet)	**M**	**4.4 (0.5)**	**137.5 (16.8)**	**31.4 (2.4)**	**−22.8 (5.5)**	**10.8 (3.7)**	**6.9 (3.6)**	**8**

*Note*: Tissues: M = muscle, B = blubber; sources: 1 = Whale, 2 = dolphin, 3 = shark, benthopelagic rays and non‐pelagic teleost, 4 = benthic rays and cephalopods, 5 = pelagic teleost, 6 = estuary‐associated teleost.

### 2.2 Sample processing and stable isotope analyses

#### 2.2.1 Tooth development and sampling

**FIGURE 2:**
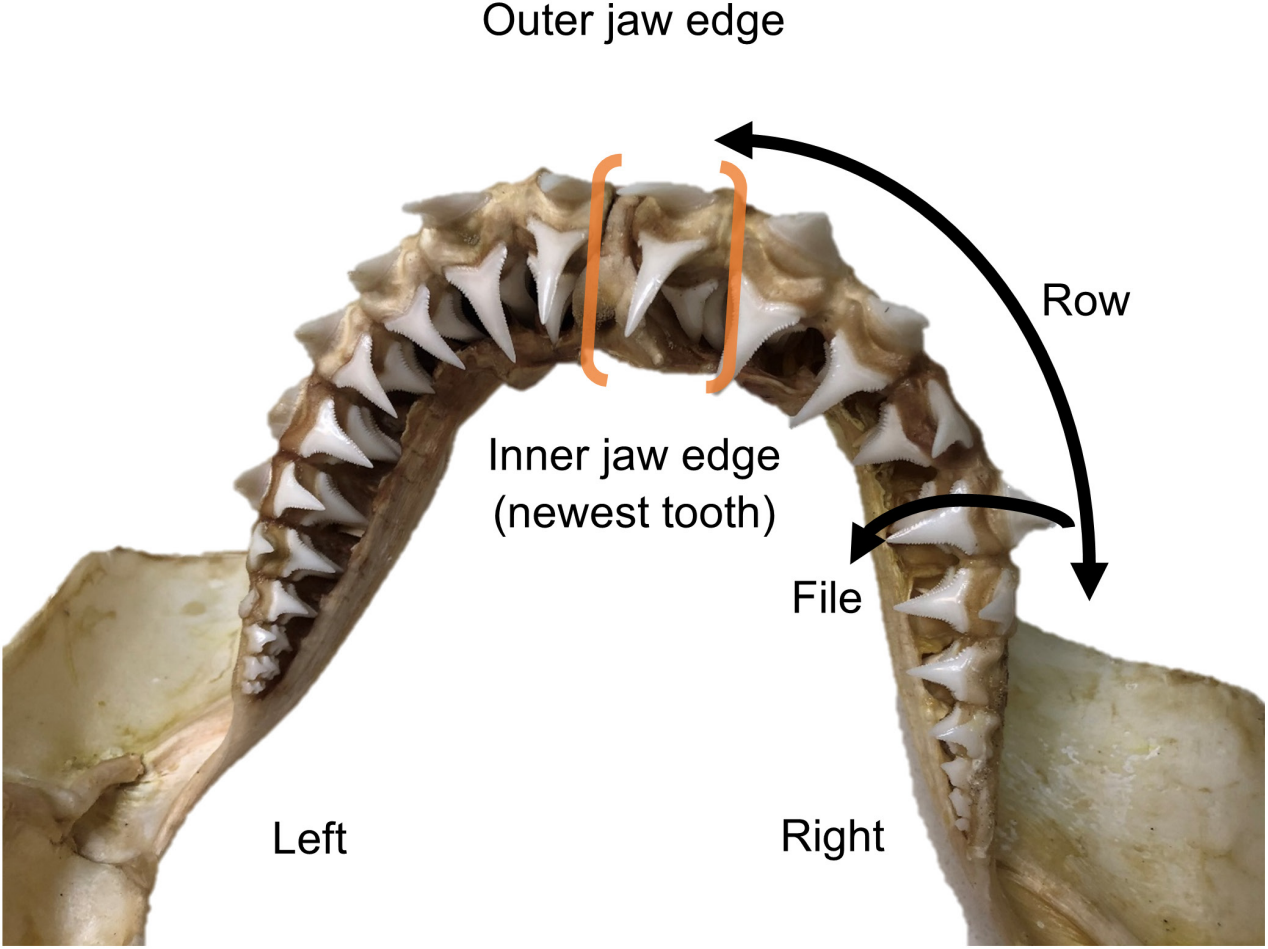
Cleaned lower jaw of a white shark (1.85 m TL) indicating dentition and descriptive terminology adapted from Becker et al. (2000). The tooth file sampled in all sharks is bracketed in orange.

Shark teeth are a composite material, comprising an outer, highly mineralised (high‐fluoride carbonated apatite) enameloid layer (~1%–8% organic protein matrix by weight; collagen and other proteins) surrounding an inner osteodentine pulp that is less mineralised (~15%–20% organic matrix; mostly collagen; Berkovitz & Shellis, 2017; Enax et al., 2012). Isotopic dietary signatures are assimilated into the organic matrix (hereafter “collagen”) during formation until the tooth becomes fully mineralised and inert, prior to eruption (Becker et al., 2000; Zeichner et al., 2017). As with other elasmobranchs, sharks develop teeth continuously below the jawline which rotate forwards in files in a conveyor belt style process to replace existing functional teeth on the outer jaw edge (Figure 2). Thereby, sampling tooth rows within a file from the inner (newest tooth) to outer jaw edge (oldest tooth) provides a sequential record of foraging patterns over temporally distinct periods (when each tooth formed) defined by rates of tooth replacement and isotopic turnover during odontogenesis (Shipley et al., 2021).

The full tooth file (5–6 rows) immediately right of the lower jaw symphysis was sampled from each white shark (Figure 2). The lower jaw was used as it generally contained more rows per file (5–6) than the upper jaw (3–4). Recent SI analyses of shark tooth collagen have indicated isotopic variability across teeth assumed to be of similar age (i.e. same row in different files; Shipley et al., 2021). Therefore, using a single file may have underestimated overall variability within the jaw. However, white sharks possess independent dentition, whereby teeth from different files can be lost and replaced at different times (Berkovitz & Shellis, 2017), complicating comparisons across files and introducing uncertainty regarding the temporal window sampled if rows (of potentially different ages) across different files are aggregated. Despite the caveat of using a single row, this preserved the assumption that rows within a file offered sequential, temporally distinct foraging information (Zeichner et al., 2017), and sampling from a consistent jaw location in all individuals minimised potential biases related to variation in rates of tooth loss/replacement in different areas of the jaw. Using the best available information (from species with greatest similarities in detention and size to white sharks, where possible), we estimated tooth replacement at 18–36 days row^−1^ (sandbar sharks *Carcharhinus plumbeus*; Wass, 1973; Table S1 for other species) and isotopic turnover (residence time) as ~32–83 days (leopard sharks, *Triakis semifasciata*; Zeichner et al., 2017). Thereby, sampled tooth files were estimated to integrate diet over ~90–216 days (depending on replacement rate and the number of available rows).

Teeth were removed using a clean knife, dried overnight (50°C) and cleaned using a colony of dermestid beetles, avoiding chemical techniques that could alter isotopic signatures. Teeth were sonicated for 5 min, triple rinsed in Milli‐Q water to remove surface contaminants, dried (50°C, 48 h) then homogenised using a ball mill (Retsch® MM 400). To remove inorganic carbon and extract the collagen matrix, ~0.4 g of powdered tooth samples were placed in centrifuge tubes with 5 ml of 0.5 M pH 8.0 ethylenediaminetetraacetic acid (EDTA; Sigma‐Aldrich®), suspended by vortex mixing (30 s) and left for 1 week. The EDTA was replaced weekly until samples were fully gelatinised (~1 month) by centrifuging (2500 rpm, 3 min) and pipetting off the supernatant. Samples were then rinsed 5 times in Milli‐Q water (vortex mixed and centrifuged between each rinse) and dried (50°C, 48 h). Whilst slow, demineralisation was performed using EDTA rather than HCl to ensure sufficient material for δ^34^S analysis (>9 mg) given the small volume of some tooth samples. Dried, demineralised samples were ball milled to a powder prior to isotope analyses.

Prey muscle and blubber were dried (50°C, 48 h) and then homogenised in a ball mill. Recent investigations suggest that lipids (and carbohydrates) may contribute significantly to proteinaceous consumer tissue isotopic signatures (predominantly δ^13^C) via de novo synthesis pathways (e.g. beta oxidation or gluconeogenesis), especially when lipids (or carbohydrates) are abundant in the diet (Arostegui et al., 2019; Newsome et al., 2014; Wolf et al., 2015). In such circumstances, using non‐lipid extracted sources has been advocated (Arostegui et al., 2019; Wolf et al., 2015). Shipley et al. (2021) noted evidence for differences in isotopic fractionation between shark tooth collagen and other tissues and suggested this was driven by a dominating de novo pathway using protein and non‐protein substrates to synthesise glycine, a non‐essential, glycolytic and principle amino acid in collagen (also see Guiry & Szpak, 2020; Whiteman et al., 2018). Considering this, and the lipid‐rich composition of some white shark prey (e.g. dolphins, whale blubber), we did not lipid extract prey samples and implemented concentration‐dependent mixing models (see below), following Wolf et al. (2015) and Arostegui et al. (2019). Additionally, to better reflect that juvenile white sharks often consume whole dolphins (Grainger et al., 2020), for which muscle and blubber are both major digestible components differing in their lipid content, weighted averages of paired muscle and blubber measurements (isotopic signatures and elemental concentrations) from each sampled dolphin individual were computed and used in mixing models. Weights were the average body mass proportions of each tissue from Mallette et al. (2016).

Approximately 9 mg of dried, powdered samples (tooth collagen and prey tissues) were weighed into tin capsules and isotopic signatures were determined using a Europa EA GSL Elemental analyser (Europa Scientific Inc., Cincinnati OH) coupled to a Hydra 20–22 automated Isoprime isotope ratio mass spectrometer (Sercon Ltd.; www.serconlimited.com) at the Griffith University Stable Isotope Laboratory, Brisbane, Queensland, Australia. Internal glycine standards with known ratios to atmospheric nitrogen, Vienna Pee Dee belemnite and Vienna‐Canyon Diablo troilite international reference standards were run (7–9 per sample tray), yielding precision (SD) of 0.1–0.2‰ for δ^15^N and δ^13^C and 0.4–0.7‰ for δ^34^S.

### 2.3 Data analyses

#### 2.3.1 Tooth collagen quality assurance and control

The amount and quality of collagen extracted from teeth was evaluated by calculating the organic matrix yield (ratio of demineralised to mineralised dry mass), and examining whether the atomic C:N ratios (C:N_atomic_) of demineralised samples fell within the recommended range of 3.0–3.3 to avoid potential isotopic effects from non‐collagenous proteins or lipids (mostly on δ^13^C; Guiry & Szpak, 2020). Organic matrix yield (mean ± SD = 14.1 ± 4.0%) was similar to that determined thermogravimetrically for shark teeth (14.7%–19.5%; Enax et al., 2012), suggesting full decalcification. In seven sharks, newly forming teeth (row 6) were present. However, these were mostly not fully mineralised and had C:N_atomic_ >3.3 (range 3.3–4.0). Given this, and to standardise sample sizes to *n* = 5 per individual for subsequent isotopic niche comparisons, row 6 samples were excluded from further analysis. Of the remaining 60 samples, 11 had C:N_atomic_ >3.3 (Figure S1). Consequently, we modelled linear relationships between C:N_atomic_ and δ^13^C, and adjusted δ^13^C for samples where a significant negative relationship (see Guiry & Szpak, 2020) was observed and C:N_atomic_ was >3.3 (*n* = 7 teeth) using a scaled offset equation approach following Shipley et al. (2021) (Figure S1). No correction was applied to an additional 7 samples with C:N_atomic_ between 2.9 and 3.0 because no obvious deviations in δ^13^C were evident (Figure S1) and the lower C:N_atomic_ limit (3.0) is conservative (Guiry & Szpak, 2020).

#### 2.3.2 Isotopic niche metrics

Individual isotopic niche specialisation was quantified using the variance component framework of BIC and WIC of variation, total niche width (TNW; BIC + WIC) and a specialisation index (s‐index; WIC:TNW; Bolnick et al., 2002; Roughgarden, 1972, 1974). These concepts were extended to a multivariate context using multiple‐response linear mixed effects modelling (MRLMM) following Ingram et al. (2018) to accommodate our trivariate isotopic data.

The MRLMM was fitted using the mcmcglmm package (Hadfield, 2010) with tooth isotopic signatures as response variables, a gaussian error distribution, individual identity as a random effect, and no fixed effects, to estimate location (i.e. mean isotopic signatures) and covariance structure parameters for the overall population. Models were fit using Markov chain Monte Carlo (MCMC) simulations (100,000 iterations, burn‐in = 3,000, thinning = 50) and convergence was checked using the Gelman‐Rubin diagnostic (coda package; Plummer et al., 2006) across five independent MCMC runs (Costa‐Pereira, Araujo, et al., 2019a). The posterior parameter means were used as the estimates for the mean and covariance matrices of the individual random effect (G‐structure, partitioning between‐individual variance, BIC_pop_) and residual error (R‐structure, partitioning remaining within‐individual variance, WIC_pop_; Ingram et al., 2018). The WIC_pop_ and BIC_pop_ covariance matrices were summed to estimate population TNW (TNW_pop_; Ingram et al., 2018). Since this modelling quantifies overall population specialisation (WIC_pop_:TNW_pop_) but does not assign specialisation metrics to particular individuals, a covariance matrix was computed for each shark (cov function; R Core Team, 2021) to estimate individuals' niche widths (WIC_ind_; see univariate analogue in Matich et al., 2011). The s‐index for each individual was defined as the “size” (sum of eigenvalues) of its WIC_ind_ matrix divided by the size of the TNW_pop_ covariance matrix (Ingram et al., 2018), scaling from 0 (small isotopic niche, noting that, in theory, it will never exactly equal 0) to 1 (large isotopic niche) as WIC_ind_ approaches the TNW_pop_.

The s‐index describes niche breadth (isotopic variation), but not overlap (overall three‐dimensional similarity) with the population, which is also an important aspect of niche differentiation. Thus, overlap of individuals with the population niche was determined using Rossman et al.'s (2016) method and code. Niches were scaled as standard ellipsoids, trivariate extensions of the bivariate standard ellipse predicted to contain ~40% of observations and representing the “core” isotopic niche of the sharks (Costa‐Pereira, Araujo, et al., 2019a; Jackson et al., 2011; Rossman et al., 2016). Overlap (o‐index) was the overlap volume between the standard ellipsoids of individuals and the TNW_pop_ standard ellipsoid, expressed as a proportion of the TNW_pop_ ellipsoid volume. O‐index values closer to 1 indicate greater three‐dimensional similarity to the TNW_pop_ ellipsoid. Combined, the s‐ and o‐indices describe the spread and displacement of individual sharks' isotopic niches, relative to the population‐level niche. To evaluate whether specialisation indices were related to shark sex or size class, several candidate generalised linear models (GLM, mgcv package with linear (non‐smooth) predictors only; Wood, 2011) were fit separately for both the s‐ and o‐indices (response variables) using a beta error and logit link (Douma & Weedon, 2019) and either sex, size class, sex + size class or a null model (intercept only) as predictors. Small‐sample corrected Akaike information criterion (AIC_c_, mumin package; Barton, 2020) was used to select the favoured model (lowest AIC_c_).

#### 2.3.3 Bayesian stable isotope mixing models

To provide ecological context to individuals' isotopic niches, proportional prey consumption by individual sharks was modelled using the MixSIAR Bayesian SI mixing model (Stock et al., 2018). The reliability and precision of these models generally decreases as the number of sources exceeds the number of isotopes +1 (four sources in our three‐isotope system; Phillips et al., 2014; Stock et al., 2018). Given the large number of known prey of white sharks (Table 1; Grainger et al., 2020), pairwise permutational analysis of variance (PERMANOVA, 9999 permutations; vegan package; Oksanen et al., 2018) tests were conducted using normalised Euclidean distances of isotopic signatures (Zintzen et al., 2013) and a Benjamini–Hochberg multiple comparisons correction (Benjamini & Hochberg, 1995) to determine whether prey did not differ significantly and could thus be grouped (Phillips et al., 2014). However, significant differences were found among most prey species (Figure S2). Consequently, mixing spaces were visually inspected and prey were grouped into six sources that had (1) general similarity in isotopic signatures, and (2) similar nutritional compositions. This provided logical groupings of species that were nutritionally similar (Figure S3), which was a priority for subsequent nutritional modelling, and were generally separated from other sources on at least one isotopic axis (Figure S4). Assigned source groupings represented the maximum level of simplification possible without excluding known important prey (Grainger et al., 2020) and thereby violating mixing model assumptions (Stock et al., 2018), or pooling nutritionally and functionally different prey and thus producing uninterpretable groups.

Since mixing models are sensitive to trophic enrichment factors (TEFs, Δ), performing sensitivity analyses to different TEFs (where they are available/applicable) is recommended (Phillips et al., 2014; Stock et al., 2018). Currently, the only experimentally measured TEFs for shark tooth collagen are from small leopard sharks *Triakis semifasciata* fed low‐lipid tilapia *Oreochromis* sp. (TEF_A_, Table 2) or squid *Loligo opalescens* (TEF_B_, Table 2) diets (Zeichner et al., 2017). However, TEF estimates from single diet items may not reflect natural scenarios where individuals consume mixed diets (Petta et al., 2020), and the appropriateness of these estimates for white sharks, which are higher trophic level predators that consume high‐lipid prey, is uncertain since both trophic level and dietary lipid content can influence trophic enrichment (Hussey et al., 2014; Newsome et al., 2014; Shipley & Matich, 2020; Wolf et al., 2015). Shipley et al. (2021) recently identified that constant isotopic offsets between tooth collagen and muscle across several larger, more ecologically equivalent shark species to juvenile white sharks, were likely driven by differences in tissue‐specific fractionation, indicative of a larger Δ^13^C and smaller Δ^15^N in teeth than muscle. Consequently, we used these offsets as quantitative proxies for the difference between muscle and tooth TEFs and thereby estimated an additional TEF (TEF_C_, Table 2) by adding (δ^13^C) or subtracting (δ^15^N) the offsets from muscle TEFs (mean Δ^13^C_muscle_ = 1.25, Δ^15^N_muscle_ = 2.78) determined for large sharks fed a bulk (non‐lipid extracted) mixed diet (Figure S5; Hussey et al., 2010), which may be more ecologically relevant for white sharks. Errors were conservatively set to the maximum standard deviations reported for Δ^13^C_teeth_ and Δ^15^N_teeth_ by Zeichner et al. (2017). Although Δ^34^S has not been determined for shark tooth collagen, generally it is negligible (Krajcarz et al., 2019; McCutchan et al., 2003), and methionine, the predominant sulfur‐containing amino acid in fish collagen (Guiry & Szpak, 2020), is essential in most animals, undergoing direct dietary routing which supports limited fractionation for sulfur (Brosnan & Brosnan, 2006; Nehlich, 2015). Therefore, Δ^34^S was set to 0.0 ± 0.5 (mean ± SD) for all TEF scenarios (Table 2) to accommodate uncertainty and measurement error (Raoult et al., 2019).

**TABLE 2:** Mean ± SD trophic enrichment factors (TEF, Δ) for the three scenarios considered for running mixing models

Scenario	Δ^13^C	Δ^15^N	Δ^34^S	Reference
TEF_A_	3.1 ± 1.0	2.8 ± 0.6	0.0 ± 0.5	Zeichner et al. (2017) (tilapia diet)
TEF_B_	4.7 ± 0.5	2.0 ± 0.7	0.0 ± 0.5	Zeichner et al. (2017) (squid diet)
TEF_C_	4.4 ± 1.0	0.9 ± 0.7	0.0 ± 0.5	Hussey et al. (2010) (muscle TEF) Shipley et al. (2021) (tooth‐muscle offset)

**FIGURE 3:**
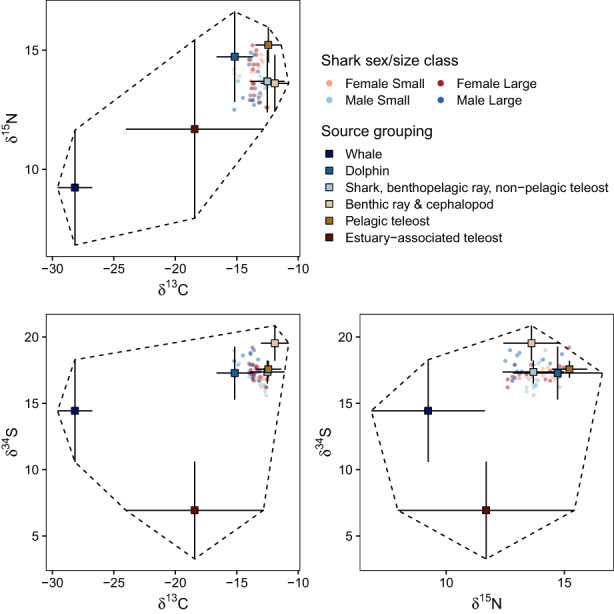
Isotopic signatures of prey source groups (squares, mean ± SD) and individual tooth samples (circles) on all three isotopic axis combinations for the TEF_C_ scenario. Prey signatures have been corrected for trophic enrichment, and errors are the combined source + trophic enrichment SD following Stock and Semmens (2016).

Mixing polyhedron simulations (3,000 iterations) were used to determine whether tooth samples fell within the 95% mixing region under each TEF scenario and thereby satisfied mixing model assumptions (Phillips et al., 2014; Smith et al., 2013). Most samples fell outside the 95% mixing region on the δ^15^N axis under TEF_A_ (Figures S6A and S7A), so this scenario was excluded. All samples fell within the 95% mixing region for TEF_B_ (Figures S6B and S7B) and TEF_C_ (Figure S7C, Figure 3), although probabilities were higher under TEF_C_ for 73.3% of samples. The Δ^13^C for TEF_C_ fell within the range of values measured in leopard sharks, but Δ^15^N was smaller (Table 2). This is consistent with observations of reduced Δ^15^N in higher trophic level species (“scaled trophic enrichment”; Hussey et al., 2014; Shipley & Matich, 2020), and the potential for additional ^15^N‐depletion when de novo protein synthesis is extensive (which is suggested for shark tooth collagen; Shipley et al., 2021) due to metabolic recycling of urea‐nitrogen retained by elasmobranchs (e.g. Whiteman et al., 2018). Given this, and the quantitative evidence for low Δ^15^N_teeth_ in ecologically similar species to white sharks upon which TEF_C_ was based (Figure S5, Shipley et al., 2021), TEF_C_ was used as the most relevant scenario for modelling white shark diets. However, parallel analyses were performed using TEF_B_ for comparison, which are provided in the Supplementary Material.

Mixing models were fitted with individual ID as a random effect (to estimate global (population) and individual‐level diets), concentration dependence (Phillips & Koch, 2002), uninformative priors and the “extreme” setting (iterations = 3,000,000, burn‐in = 1,500,000, thinning = 500, chains = 3) to ensure convergence, which was validated with the Gelman‐Rubin diagnostic. This produced 9,000 posterior vectors of prey proportions (each vector summing to 1) for each individual and the population. Informative priors based on stomach contents studies were not included since these represent only population‐level patterns that could bias results for individuals whose isotopic/dietary patterns differed from the population (e.g. Swan et al., 2020).

To quantify individual specialisation (differences from the population) in p‐space, the mean cosine similarity (c‐index_prey_; lsa package; Wild, 2020) between individuals and the population across all 9,000 posterior prey proportion vectors was computed following Newsome et al. (2012). The c‐index varies between 0 and 1, with dissimilarity from the population increasing as c‐index → 0. To identify the sources driving differences from the population, probabilistic comparisons between each individual and the population were computed (proportion of posterior estimates for individual *j* < population) as a proxy for the probability that prey posterior distributions differed (see Jackson et al., 2011; Manlick et al., 2019; Stock & Semmens, 2016). Differences were inferred for probabilities >0.95 (*j* < population) or <0.05 (*j* > population). Differences between mixing model outputs based on TEF_C_ and TEF_B_ were also assessed using this method.

#### 2.3.4 Nutritional modelling using mixing model posterior outputs

To evaluate the nutritional outcomes of diet variation estimated by the mixing model, we combined prey nutritional values with the posterior proportional source contributions. Macronutrient compositions (wet mass % water (%W), lipid (%L), protein (%P); carbohydrates excluded as they are negligible in most marine prey; Craig et al., 1978) were obtained from the literature for prey species included in the source groupings (Table S2; see Data Sources section; also see Grainger et al., 2020). Compositions were extracted, where possible, from studies conducted in geographical proximity to the present study (Tait et al., 2014), and values for closely related taxa (same genus/family) were used if compositions were unavailable for particular prey species (Table S2; Eder & Lewis, 2005). Compositions of prey species were generally similar within source groupings (Table S2, Figure S3) and mean proximate compositions were used where multiple species were aggregated into a single source.

For each posterior prey proportion vector *i* (representing relative prey biomass assimilation; Phillips & Koch, 2002), we calculated the % dietary nutrient intake with respect to the nutrient components %P, %L and %W using

$$\%Y_{i,\text{diet}}=\sum_{t=1}^{n}\%Y_{t}\times P_{i,t},$$

where %*Y*_*t*_ is the % of nutrient *Y* in source *t*, *P*_*i*,*t*_ is the dietary proportion for source *t* at iteration *i* and %*Y*_*i*,diet_ is the % of nutrient *Y* in the diet at iteration *i*. This transformation generated posterior distributions of dietary nutrient intakes with uncertainty propagated from the prey proportion posteriors estimated by the mixing model. Prey and dietary nutrient compositions (posterior means ± SD) for each shark were visualised using graphical proportions‐based NGF models (Raubenheimer, 2011). These plot three proportional nutrients in two dimensions, here %P (*x*‐axis), %L (*y*‐axis) and %W, which sum to 100% so that the combined %P + %L value implies %W, which increases towards the origin (as %P and %L decrease). A mean ± SD dietary nutritional intake estimated for juvenile white sharks from stomach contents (%W_SCA_, %P_SCA_, %L_SCA_, P:L_SCA_; *n* = 40; Grainger et al., 2020) was also plotted for comparison with SI estimates (%W_SI_, %P_SI_, %L_SI_, P:L_SI_).

Individual specialisation in N‐space was assessed using cosine similarities (c‐index_nutrients_) and probabilistic comparisons of individuals with the population, as above for p‐space estimates. Since dietary nutrient intakes were naturally restricted to the limits of the prey nutrient compositions (e.g. 1.3%–45.0% lipid, instead of 0–100% lipid), the c‐index_nutrients_ was rescaled to ensure the lower bound (c‐index_nutrients_ = 0) represented the minimum possible cosine similarity value for the given range of prey compositions. Candidate beta GLMs (Douma & Weedon, 2019) were fit and compared via AIC_c_ to investigate possible effects of sex, size class or sex + size class (predictors) on c‐index_prey_ and c‐index_nutrients_ (response variables), as for s‐ and o‐indexes above. The relationship between c‐index_prey_ (predictor) and c‐index_nutrients_ (response) was also evaluated using a beta GLM to test whether similar levels of individual specialisation (i.e. differences from the population) were maintained by individuals across different niche spaces. A positive relationship was predicted in this circumstance. All data analyses were conducted in R (v4.2.0; R Core Team, 2021), and figures were generated using ggplot2 (Wickham, 2016). Where relevant, results are reported as means ± SD unless otherwise indicated.

## 3 RESULTS

### 3.1 Isotopic niche metrics

**FIGURE 4:**
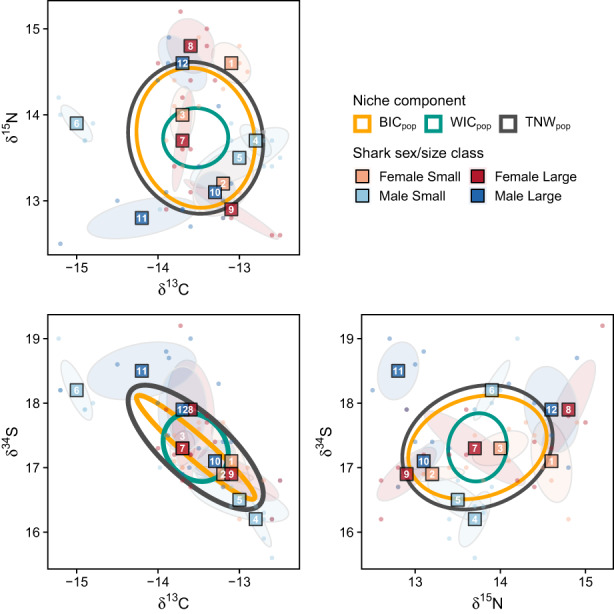
Two‐dimensional projections of tooth collagen isotopic signatures (δ^13^C, δ^15^N, δ^34^S) from 12 white sharks. Dots show isotopic values for individual teeth. Squares and filled ellipses show means and standard ellipses for each individual, with ID labels corresponding to those used in Figure 1. Note that means for sharks 3 and 7 overlap on the δ^13^C‐δ^34^S axis. Standard ellipses corresponding to population‐level estimates for the between‐individual (BIC_pop_) and within‐individual (WIC_pop_) components of variation and total niche width (TNW_pop_) are also shown.

**FIGURE 5:**
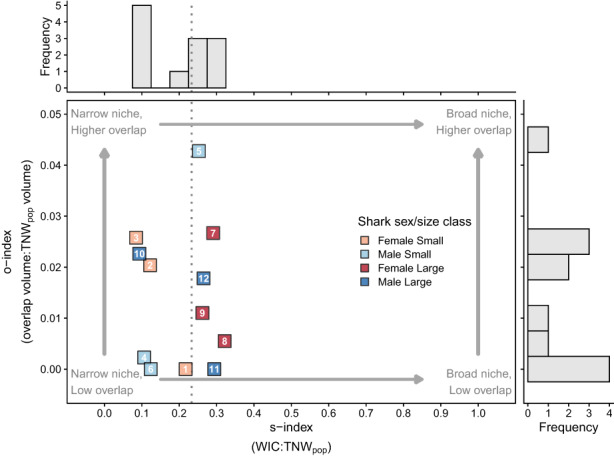
Distributions of specialisation (s‐index) and overlap (o‐index) indices for the isotopic niches of individual white sharks. The s‐index measures the breadth of individuals' isotopic niches (WIC) as a proportion of total population niche breadth (TNW_pop_). The dashed vertical line is the overall s‐index (WIC_pop_:TNW_pop_) estimated from a multiple response random effect model. Points are the s‐indexes computed at the individual level (WIC_ind_:TNW_pop_). The o‐index measures the 3‐dimensional overlap of individuals' standard ellipsoids with the population standard ellipsoid, proportional to the population ellipsoid volume. Individual ID labels correspond to those provided in Figure 1.

Across all sharks, tooth collagen isotopic signatures ranged from −15.2 to −12.5 (mean ± SD = −13.5 ± 0.7‰) for δ^13^C, 12.5 to 15.2 (13.7 ± 0.7‰) for δ^15^N and 15.6 to 19.2 (17.3 ± 0.8‰) for δ^34^S (Figure 4). However, individuals' isotopic niches occupied only subsets of these ranges, with high among‐individual variation and no clear groupings in δ‐space according to size and sex (Figure 4). For example, some biologically “similar” individuals (age, sex, capture date and location; Figure 1) occupied different isotopic niches (e.g. ws8 and ws9 (large females), and ws11 and ws12 (large males)), suggesting temporally consistent differences in foraging patterns (Figure 4). The s‐index (WIC_pop_:TNW_pop_) supported that individuals had narrow isotopic niches compared with the population, and between‐individual variation explained a greater percentage of total population variation (BIC_pop_:TNW_pop_ = 76.7%) than within‐individual variation (WIC_pop_:TNW_pop_ = 23.3%, Figure 5). Individual‐level s‐indexes (WIC_ind_:TNW_pop_) revealed variation in relative niche breadth among individuals (Figure 5). This was best explained by shark size (Table S3), with larger sharks having significantly broader isotopic niches (i.e. closer to the total population niche breadth; beta GLM, est_large.sharks_ ± SE = 0.6 ± 0.3, z = 2.3, *p* = 0.019, Figure 5). Nonetheless, most individuals had low overlap (o‐index) with the overall population niche (Figures 4 and 5) consistent with individual specialisation (i.e. low similarity to the population). Some individuals had higher s‐indexes but low o‐indexes (e.g. ws8, ws11) indicating relatively broad, but displaced niches compared with the overall population (Figures 4 and 5). O‐index values were not related to sex or size class, with AIC_c_ favouring a null model (Table S3).

### 3.2 Stable isotope mixing models

#### 3.2.1 Population‐level estimates (TEF_C_ )

**FIGURE 6:**
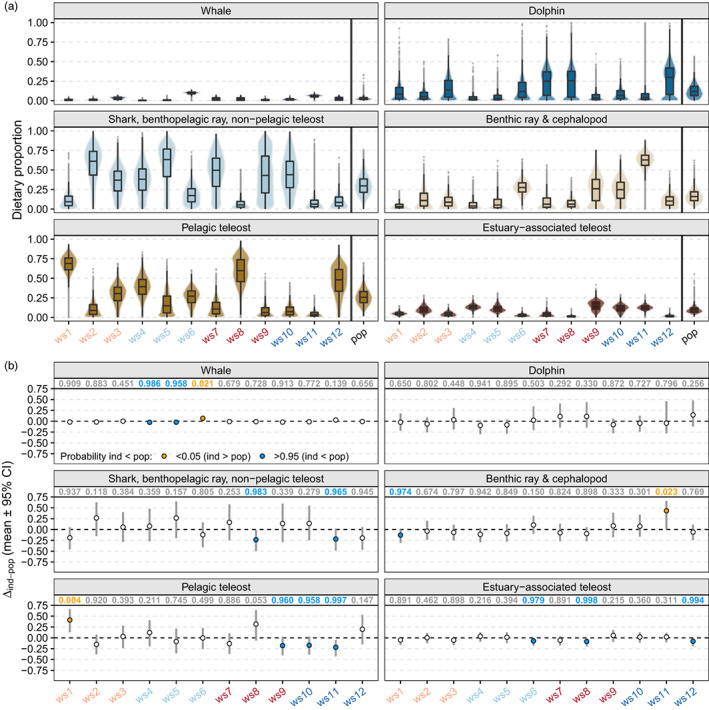
(a) Violin plots of posterior distributions for prey contributions to the diets of individual white sharks (ind, ws1–ws12) and the overall population (pop) under the TEF_C_ scenario. (b) Mean and 95% credible intervals (CI) of differences between estimated prey proportions of individuals and the population (Δ_ind‐pop_). The probabilities that ind < pop are indicated along the top of each plot for each prey source. Differences were inferred for probabilities >0.95 (ind < pop) or <0.05 (ind > pop). Individual shark labels are colour coded for sex and size as in other figures.

Prey contributions for the overall population were greatest for the shark, benthopelagic ray and non‐pelagic teleost group (mean ± SD = 30.5 ± 12.3%), followed by pelagic teleosts (26.3 ± 10.4%), benthic rays and cephalopods (16.9 ± 8.3%), dolphins (13.4 ± 9.0%), estuary‐associated teleosts (9.8 ± 4.7%) and whale (3.1% ± 2.2%, Figure 6a).

#### 3.2.2 Individual‐level estimates (TEF_C_ )

**FIGURE 7:**
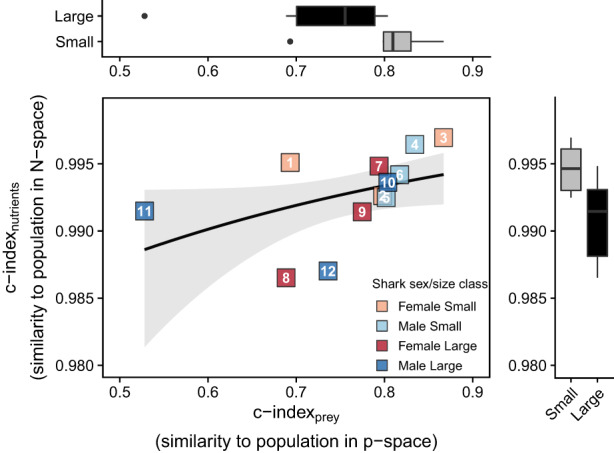
Posterior mean cosine similarities (c‐index) between individual white sharks and the overall population based on modelled prey proportions (c‐index_prey_, p‐space) and nutrient intakes (c‐index_nutrients_, N‐space) under the TEF_C_ scenario. Marginal boxplots compare variation in c‐index_prey_ (top) and c‐index_nutrients_ (right) among small (~1.50 m PCL, *n* = 6) and large (~2.25 m PCL, *n* = 6) size classes. The predicted relationship (shading = 95% confidence intervals) between the c‐index_prey_ and c‐index_nutrients_ was not significant (beta GLM, *p* = 0.061) but is shown to illustrate the deviation of some individuals (e.g. ws1, ws11) from the expected positive relationship.

Although posterior distributions were variable for some sources/individuals (e.g. shark, benthopelagic ray, non‐pelagic teleost, dolphins), significant deviations of individuals from the population (Δ_ind‐pop_) were still detected for all sources except dolphins, and in all individuals except ws2, ws3 and ws7 (Figure 6a,b). These deviations were small for whale and estuary‐associated teleost groups (Δ_ind‐pop_ <10% for all individuals), with greater individual variation in the use of shark, benthopelagic ray and non‐pelagic teleost, benthic ray and cephalopod, and pelagic teleost source groups (Figure 6b). For these sources, significant Δ_ind‐pop_ mostly ranged between (mean ± SD) ‐12.7 ± 7.9% (ws1, benthic rays and cephalopods) and ‐23.5 ± 12.5% (ws8, shark, benthopelagic rays, non‐pelagic teleosts group) from the population (Figure 6b). Individuals ws11 and ws1 displayed greater Δ_ind‐pop_, with higher contributions from benthic rays and cephalopods (+43.3 ± 14.5%) and pelagic teleosts (+41.1 ± 13.5%), respectively (Figure 6b). Individual ws8 was also comparatively specialised with high contributions of pelagic teleosts (+31.6 ± 18.0%, Figure 6a) relative to the population, although the probability for this difference was not significant (Figure 6b). Although there was a trend towards larger sharks being more dissimilar to the population in p‐space (c‐index_prey_, Figure 7), AIC_c_ favoured a null model (no sex or size effects, Table S4). Interestingly, several individuals (e.g. ws1, ws8, ws11) with comparatively broader isotopic niches (higher s‐index, Figure 5), had specialised diets (Figure 6a) dissimilar to that of the population (low c‐index_prey_, Figure 7), whilst some individuals with narrow isotopic niches (e.g. ws3, Figure 5) maintained broader diets and high similarity to the population (Figures 6A and 7).

### 3.3 Nutritional modelling

#### 3.3.1 Population‐level estimates (TEF_C_ )

**FIGURE 8:**
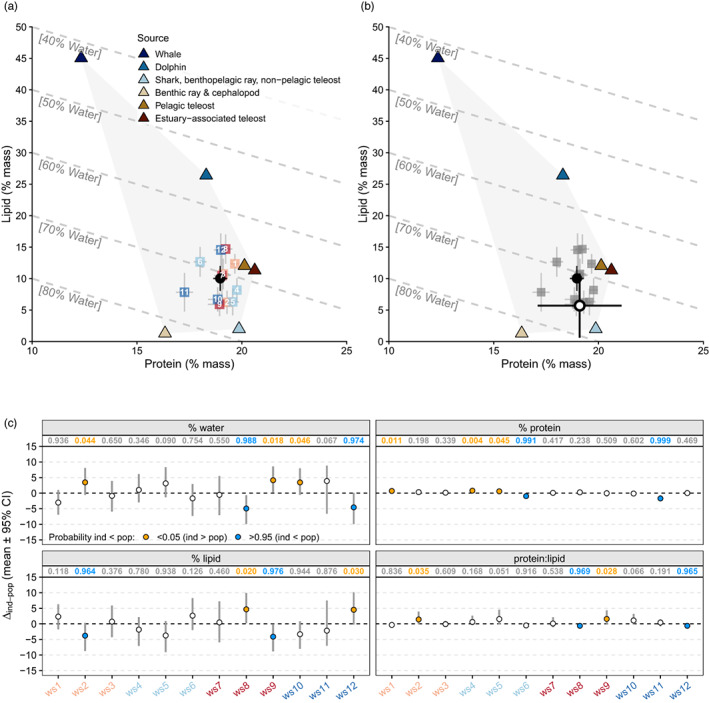
(a) Proportion‐based nutritional geometry framework model of the wet mass % of protein, lipid and water in prey sources (triangles) and diets (posterior mean ± SD) of individual white sharks (squares) and the overall population (black circle) under the TEF_C_ scenario. (b) Mean ± SD nutrient intake for juvenile white sharks based on stomach contents (white circle, *n* = 40; Grainger et al., 2020) overlayed on mixing model estimates (grey squares = individuals, black circle = population) for comparison. (c) Mean ± 95% credible intervals (CI) of differences between nutrient intakes of each white shark (ind, ws1–ws12) and the overall population (pop, Δ_ind‐pop_). The probabilities that ind < pop are displayed along the top of each plot for each nutritional variable. Differences were inferred for probabilities >0.95 (ind < pop) or <0.05 (ind > pop). Individual sharks are labelled and colour coded for sex and size as in other figures.

The estimated population‐level nutrient intake was (mean ± SD) 71.0 ± 2.0%W_SI_, 19.0 ± 0.3%P_SI_ and 10.0 ± 2.0%L_SI_ with a P:L_SI_ of 2.0 ± 0.4 (Figure 8a). Population‐level estimates from SI were generally similar to and fell within 1 SD of mean estimates based on stomach contents (75.2 ± 5.9%W_SCA_, 19.1 ± 2.0%P_SCA_, 5.7 ± 5.1%L_SCA_, P:L_SCA_ = 7.9 ± 8.3, Figure 8b).

#### 3.3.2 Individual‐level estimates (TEF_C_ )

Significant Δ_ind‐pop_ in nutrient intakes were detected for all individuals, except ws3 and ws7, with the greatest differences being on the %L_SI_ and %W_SI_ axes (Figure 8c). Nonetheless, the magnitude of deviation from the population was small overall (mean Δ_ind‐pop_ <5% for all nutrients and individuals, Figure 8c), compared with the ranges possible given the prey nutrient compositions (e.g. 42.6–82.4%W, 12.3–20.6%P, 1.3–45.0%L, Figure 8a). High similarity in nutrient intakes was also evident in the c‐index_nutrient_ values, which were close to 1 for all individuals (Figure 7). Larger sharks had significantly lower c‐index_nutrients_ (beta GLM; est_large.sharks_ ± SE = −0.5 ± 0.2, *z* = −2.6, *p* = 0.010), indicating greater dissimilarities in nutrient intakes from the population (Figure 7, Table S4). There was no significant relationship between c‐index_prey_ and c‐index_nutrients_ (beta GLM; est_c‐index.prey_ ± SE = 2.0 ± 1.1, *z* = 1.9, *p* = 0.061), suggesting that specialisation across p‐space and N‐space were not consistently correlated (Figure 7). This was predominantly driven by individuals ws1 and ws11 which showed low p‐space similarity (c‐index_prey_) but higher N‐space similarity (c‐index_nutrients_), on par with that of individuals who were less specialised in p‐space (Figure 7).

### 3.4 Comparison between TEF_C_ and TEF_B_

Sensitivity analyses suggested similarities but also some differences in model outputs between TEF scenarios, which were more pronounced in p‐ than N‐space. Proportions of dolphin, benthic ray and cephalopod and estuary teleost sources tended to be higher under TEF_B_ for both individual and population estimates, although aside from estuary teleosts for ws2, these differences were not significant (probabilities <0.95 and >0.05; Figures S8 and S9A). The most pronounced differences were in contributions from pelagic teleost, which were significantly lower under TEF_B_ for ws1 (ΔTEF_B_‐TEF_C_ = −54.5 ± 20.5%), ws6 (−21.1 ± 12.0%), ws8 (−47.9 ± 23.1%) and the population (−20.2 ± 12.1%; Figures S8 and S9A). No significant differences in modelled nutrient intakes between TEF scenarios were detected (Figures S9B and S10). As with TEF_C_, no significant relationship between c‐index_prey_ and c‐index_nutrients_ was detected under TEF_B_ (beta GLM; est_c‐index.prey_ ± SE = 3.7 ± 2.0, *z* = 1.8, *p* = 0.069), driven again by a low c‐index_prey_ and high c‐index_nutrients_ for ws1, ws11, and also ws4 (Figure S11). Comparisons with AIC_c_ favoured no effect of sex or size on c‐index_prey_ or c‐index_nutrients_ under TEF_B_, although a trend towards larger sharks having lower c‐index_nutrients_ was evident (Table S4, Figure S11), similar to the statistically significant pattern identified for TEF_C_ (Figure 7).

## 4 DISCUSSION

We have presented a framework unifying SI and nutritional geometry that addresses the challenge of examining time‐integrated nutrition in wild populations and revealed interrelationships in dietary specialisation across three key metrics of dietary niches (isotopes, prey and macronutrients) and two ecological levels (individuals and population) in white sharks. We identified a broad population‐level foraging niche (δ‐ and p‐space), comprised of individuals that used subsets of specific prey in different proportions to the overall population. Deviations from the population in nutrient intakes (N‐space) were comparatively small, and not consistently correlated with deviations in prey use. Combined together, our results reveal interesting differences in dietary specialisation depending on the ecological level or niche space assessed which have important extrinsic (ecological) and intrinsic (physiological) implications, and highlight some conceptual limitations of inferring individual diet specialisation from isotopic variance alone. We first discuss inferences on prey use based on δ‐ and p‐space analyses, then how these relate to nutritional outcomes.

### 4.1 δ‐space and p‐space specialisation: Patterns and discrepancies

Isotopic and p‐space analyses characterised white sharks as prey‐use generalists at a population level, but prey‐use specialists at an individual level. Indeed, total isotopic variability was similar to that previously established for other generalist species in the region (e.g. tiger sharks, *Galeocerdo cuvier*; Ferreira et al., 2017) and, consequently, a broad mix of prey sources comprised the population‐level diet. Movement between environmental isotopic baselines can also contribute to consumer isotopic variation, and thus may affect dietary inferences from SI (Ramos & Gonzalez‐Solis, 2012; Shipley & Matich, 2020). However, surveys indicate minimal baseline variability in δ^13^C and δ^15^N over the core range of our sampled white shark population (coastal shelf habitats off eastern Australia between 28–38°S; Raoult et al., 2020; Revill et al., 2009; Spaet et al., 2020), supporting diet as a likely primary driver of the patterns we observed. While there was some variation in diet estimates under the alternate TEF_B_ scenario (predominantly for pelagic teleosts), the TEF_C_ scenario was the most appropriate for our system (as per rationale in the Methods). TEFs remain an uncertainty in the use of mixing models, and further experimental determinations of isotopic fractionation and turnover for teeth in elasmobranchs, including the potential influence of dietary lipid content or mixed vs single‐item diets (Petta et al., 2020; Wolf et al., 2015), is a priority. Additionally, our mixing space necessitated the use of some generalised prey categories which restricted our ability to discern amongst certain prey (e.g. sharks, benthopelagic rays and non‐pelagic teleosts). However, the overall modelled population‐level importance of pelagic eastern Australian salmon *Arripis trutta* combined with a mix of sharks, benthopelagic and benthic rays and non‐pelagic teleosts is corroborated by similar findings from stomach contents (Grainger et al., 2020; Hussey et al., 2012; Tricas & McCosker, 1984), SI and fatty acid tracers (Pethybridge et al., 2014; Tamburin et al., 2020) for juvenile white sharks. The relatively infrequent consumption for dolphins, and especially whales, is also consistent with previous studies (Grainger et al., 2020; Hussey et al., 2012). Moreover, prior stomach content information helps to clarify some distinctions among prey that were pooled, notably the likely importance of benthic rays compared with cephalopods (Grainger et al., 2020).

The ecological consequences of individual diet specialisation can depend on the magnitude and specific nature of the trophic interactions that vary between individuals (Araujo et al., 2011; Bolnick et al., 2002; Ingram et al., 2011). However, many isotopic studies assess individual diet specialisation in δ‐space alone and thus do not offer specific information on individual differences in food use (Matich et al., 2021; Shipley & Matich, 2020). We addressed this limitation using mixing models, which offered improved insights into individual variation in the likely ecological functional roles of a top marine predator. Specifically, our findings suggested that individual white sharks differed predominantly in their use of pelagic eastern Australian salmon, shark, benthopelagic and benthic rays and non‐pelagic teleosts. These differences could result from resource partitioning (e.g. to mitigate competition; Bolnick et al., 2002; Matich & Heithaus, 2014), or be emergent from intrinsic attributes like hunting mode preferences (Papastamatiou et al., 2022; Towner et al., 2016). Short‐term spatiotemporal heterogeneity in prey availability could also contribute to among‐individual differences, given individuals' tooth files covered subsets (<1 year) of the timescale over which most samples were collected (~5 years), although vertebral SI in white sharks supports the likely long‐term persistence of individual specialisations across varying ecological contexts (Kim et al., 2012). The drivers of among‐individual variation warrant further investigation. However, our findings nonetheless indicate individual variation in predation pressure among white sharks, persisting over significant spatiotemporal scales (3–6 months integrated by teeth files), on several prey species which are themselves important predators in pelagic (e.g. Australian salmon; Hughes et al., 2014) and/or benthic food webs (e.g. hammerhead sharks (benthic/pelagic) or rays (benthic); Flowers et al., 2021; Gallagher & Klimley, 2018; Myers et al., 2007). This not only suggests functional inequivalence among individual white sharks but identifies specific trophic routes through which this may arise; in particular, via individual‐specific top‐down pressure in either benthic or pelagic systems.

Establishing individual‐level diets with mixing models also highlighted some important differences between δ‐ and p‐space with wider implications regarding the conceptualisation and interpretation of δ‐space variance as a proxy for individual diet specialisation. Specifically, although larger sharks had broader relative isotopic niche breadths (larger s‐index) and could be inferred to exhibit greater dietary generalism, this was not supported in p‐space. Rather, prey use by larger individuals tended to be more dissimilar to the overall generalist population (lower c‐index_prey_), although this was not significant. Nevertheless, several individuals with the broadest isotopic niches exhibited specialised diets (e.g. ws1, ws8, ws11) whilst others with narrow isotopic niches had broader diets, similar to the population (e.g. ws3). This may be explained by the fact that isotopic niche size does not necessarily correlate with niche overlap, which is itself of chief relevance to dietary partitioning (Hette‐Tronquart, 2019). The broad (high s‐index) yet displaced (low o‐index) isotopic niches of some sharks supported this. More generally, consumer isotopic variation arises from potentially confounding interplays between feeding strategies, their temporal scales, and mixing space geometry (Hette‐Tronquart, 2019; Yeakel et al., 2016). For instance, a generalist may still exhibit a narrow isotopic niche by consistently using the same wide mix of prey and thereby averaging their isotopic signatures, whilst a broad isotopic niche may result either from temporal switching between many prey sources or through moderate specialisation on a few sources with comparatively disparate signatures in the mixing space (Hette‐Tronquart, 2019; Yeakel et al., 2016). Overall, our results highlight how the relative isotopic variance of individuals (as per applications of the classical WIC:TNW framework in δ‐space, our s‐index) alone may not provide a reliable proxy for degrees of individual specialisation in terms of actual food use. Mixing models may assist in accurately identifying individual diet specialisations because this approach holistically evaluates how the combined variance, overlap and position of individuals' isotopic niches relates to, and arises from, the isotopic distributions of the resources they consume.

### 4.2 From p‐space to N‐space: Integrating a nutritional dimension of individual diet specialisation

Establishing flexibility in the nutritional niche of individuals, populations and species is paramount for understanding physiological bases of animals' responses to variation in their environment (Machovsky‐Capuska, Senior, et al., 2016b; Simpson & Raubenheimer, 2012), although this is challenging to achieve in research involving free‐ranging species, especially cryptic predators (Machovsky‐Capuska, Coogan, et al., 2016a). Our integrative framework offers a new solution for this, and similarities between nutritional estimates from SI and stomach contents for white sharks highlights the efficacy of our approach. A consideration of our framework is that if different foods are pooled due to isotopic similarity, they are assumed to be representable by their overall mean nutritional composition for subsequent nutritional modelling. In our study, this assumption was justified because species within the isotopically pooled groups were nutritionally similar. This assumption may be met within marine systems more broadly because species within certain taxonomic/trophic groups (which are likely to be isotopically similar) exhibit characteristic nutritional profiles (e.g. low protein/lipid in rays and many cephalopods, high lipid in pelagic forage fish; Eder & Lewis, 2005; Spitz et al., 2010; Vollenweider et al., 2011). In terrestrial systems, body protein and lipid have been shown to covary with trophic level (δ^15^N) in some taxa (Wilder et al., 2013). While further exploration of such relationships would be beneficial, this supports the likelihood of congruent nutritional properties within broad taxonomic/trophic groups, which are generally best suited for use in mixing models anyway (e.g. Manlick et al., 2019; Semmens et al., 2009).

Important findings revealed by our framework were that individual white sharks varied little from the population in N‐space (<5% for all nutrients, c‐index_nutrients_ close to 1), and that individuals which differed in prey use (e.g. ws1, ws11) did not necessarily show commensurate deviations from the population in N‐space, leading to no significant relationship between p‐ and N‐space specialisation. Combined, this suggests white sharks are nutritional specialists at both the individual and population level and highlights how food use variation or partitioning may not be reflected similarly in the nutritional dimension, with differing ecological and physiological implications. For instance, nutritional specialists may respond more strongly (behaviourally or physiologically) to fluctuations in their nutritional environment, whilst nutritional generalists can tolerate and thereby persist across a wider array of contexts (Machovsky‐Capuska, Senior, et al., 2016b; Senior et al., 2016). Predators often vary in their food use between individuals and/or populations (Manlick et al., 2019; Newsome et al., 2012; Semmens et al., 2009; Stock et al., 2018; Votier et al., 2010). However, the nutritional and physiological implications of this, and how patterns of food use variation may depend on flexibility in requirements for particular nutrients (the degree of N‐space specialisation), remain largely unexplored (but see Remonti et al., 2016). Elucidating such dynamics requires considering the functional relationships between different foods, which may offer similar nutritional properties (“substitutable” feeding), or be nutritionally different and imbalanced, yet able to be combined in proportions necessary for a specific nutritional goal (“complementary feeding”; Behmer et al., 2001; Raubenheimer, 2011; Raubenheimer & Jones, 2006; Simpson & Raubenheimer, 2012). Nutrient balancing through complementary feeding has been documented in many species (herbivores, omnivores, carnivores) in the laboratory (Simpson & Raubenheimer, 2012), and leveraged to infer nutritional priorities (targeted macronutrient balance) in field‐based settings, although predominately in primates (Hou et al., 2021; Raubenheimer et al., 2015). Knowledge on whether similar mechanisms operate in free‐ranging carnivores remains limited (Kohl et al., 2015; Machovsky‐Capuska, Coogan, et al., 2016a). Most of the prey sources on which white sharks differentiated their diets were nutritionally distinct. Thus, maintenance of similar intakes to the overall population for some individuals (e.g. ws1, ws11), despite widely differing prey use, could suggest complementary feeding mechanisms in white sharks. Overall, these findings highlight the utility of our approach for elucidating nutritional specialisation/generalism and mechanisms of nutrient‐specific foraging (e.g. substitutable vs. complementary feeding) in species where time‐integrated foraging observations are otherwise impossible. Although we applied this in the context of individual diet specialisation using serially sampled tissues, mixing models can estimate individual‐ and population‐level diets from single samples (albeit without accommodating WIC; Manlick et al., 2019; Stock et al., 2018) and comparing diet variation in p‐ vs N‐space across individuals or populations can offer insights into animals' nutritional priorities (see Raubenheimer et al., 2015; Remonti et al., 2016).

Despite the overall similarity of individuals in N‐space, significant deviations in nutrient intakes were still detected, especially for larger sharks, which could potentially reflect varying macronutrient preference or physiological requirement (e.g. Grainger et al., 2020; Han et al., 2016). Alternatively, these differences could indicate nutritional constraints, which may have fitness or performance consequences (Simpson & Raubenheimer, 2012). Indeed, fitness and performance parameters have been related to increased isotopic variance (Costa‐Pereira, Toscano, et al., 2019b) and individuals' use of specific foods (Robertson et al., 2015; Votier et al., 2010), but the physiological basis for these relationships remains unclear. Adopting a nutrient‐specific approach to modelling foraging can directly link food use to fitness/performance (Jensen et al., 2012), and we suggest that expanding our approach by relating time‐integrated nutrient intakes (estimated via SI) to fitness/performance proxies measured on the same individuals (e.g. performance surfaces mapped over N‐space; Simpson et al., 2004) could establish such links in field‐based contexts. Doing so would help better define animals' fundamental dietary niches as the breadth of nutrient intakes over which performance/fitness is maintained, which is critical for understanding individual/species' resiliencies to environmental variation or disturbance (Machovsky‐Capuska, Senior, et al., 2016b; also see Takola & Schielzeth, 2022).

## 5 CONCLUSIONS

We have presented a unification of stable isotopes and nutritional geometry that simultaneously evaluated individual specialisation across isotopic, prey use and nutritional niches and distinctions among these, in juvenile white sharks. By revealing individual‐level differences in prey use, our findings highlight the potential variable trophic roles played by individual white sharks in either benthic or pelagic food webs. Additionally, modelling individual‐level diets demonstrated the limitations of inferring individual diet specialisation from isotopic niche size alone, which is a product of multiple, complicating factors in addition to diet breadth. Extending our analysis into nutrient space suggested white sharks as nutritional specialists at both the individual and population level, and the potential for specialisation on different prey that provide complementary means of achieving a similar nutritional goal. While we applied our framework using the sequential dentition of elasmobranchs, the method could equally be applied in other systems (e.g. other serially accreted tissues like hair/whiskers, tissues with different turnover rates,or repeated sampling of individuals where feasible; Newsome et al., 2012; Semmens et al., 2009; Votier et al., 2010). Integrating a nutritional dimension of individual diet specialisation could help better define nutritional generalism and establish mechanistic links, formulated around macronutrient balance, between individual fitness, foraging specialisation and its ecological outcomes. The wider adoption of nutrient‐specific approaches is a priority in trophic ecology (Danger et al., 2022) and linking SI with nutritional geometry more generally holds potential for a range of important questions, such as understanding the nutritional consequences (or drivers) of food use variation between populations (e.g. Manlick et al., 2019) or under a changing climate (e.g. Young et al., 2015).

## AUTHOR CONTRIBUTIONS

Richard Grainger, Victor M. Peddemors, Gabriel E. Machovsky‐Capuska and David Raubenheimer conceived the ideas and design of the study. Richard Grainger and Victor M. Peddemors collected the samples. Richard Grainger and Vincent Raoult processed the samples and collected the data. Richard Grainger analysed the data. Richard Grainger led the writing of the manuscript and all authors contributed critically to the drafts and gave final approval for publication.

## CONFLICT OF INTEREST

The authors declare no conflict of interest.

## ETHICS STATEMENT

No animals were killed specifically for this research. All samples were collected from animals already caught and deceased in commercial fishing operations, government‐operated bather protection programs, fisheries compliance seizures or, in the case of whales, from deceased, stranded animals. This research was performed under New South Wales Department of Primary Industries (NSW DPI) permits P01/0059(A)‐4.0 and P01/0059(A)‐2.0, and NSW DPI Animal Research Authority 07/03. Additional permits through the University of Sydney or the University of Newcastle were not required.

## Supporting information


Data S1

## Data Availability

Data are available from the Dryad Digital Repository https://doi.org/10.5061/dryad.h44j0zppd (Grainger et al., 2022).
