# Supplementary material for: Integrating isotopic and nutritional niches reveals multiple dimensions of individual diet specialisation in a marine apex predator
Source: J Anim Ecol. 2022 Dec 23;92(2):514–34. doi: 10.1111/1365-2656.13852 (PMC10107186; doi:10.1111/1365-2656.13852)
Supplement: Supplementary file 1 — Data S1 [file JANE-92-514-s001.docx]

**Table S1.** Tooth replacement rates (period between replacements of successive rows) for shark species reported in the literature. Replacement rates are those estimated directly through captive experiments and are given as days per row, unless otherwise indicated.

| **Species** | **Common Name** | **Replacement rate** | **Length/ weight / Life Stage** | **Jaw Measured** | **Method** | **Study Duration** | **Time of Year** | **Reference** |
| --- | --- | --- | --- | --- | --- | --- | --- | --- |
| *Mustelus canis* | Smooth Dogfish | 10-12 | 36.8-124.5 cm | Lower | Tooth removal & marking | 25 days | Summer | (Ifft & Zinn, 1948) |
| *Heterodontus francisci* | Horn shark | 3-4 weeks | - | - | - | - | - | (Applegate, 1967) |
| *Negaprion brevirostris* | Lemon shark | 8-10 | - | - | Marked teeth | 23 days | Summer | (Moss, 1967) |
| *Scyliorhinus canicula* | Small-spotted catshark | 5 weeks | - | - | - | 12 weeks | Summer | (Markel & Laubier, 1969) |
| *Negaprion brevirostris* | Lemon shark | 14 | Young (3-5 kg) | - | Tetracycline marking | 2-4 weeks | Summer | (Boyne, 1970) |
| *Carcharhinus plumbeus* | Sandbar shark | 18 | Immature (50 cm) | Upper | Marked teeth | 5-6 weeks | - | (Wass, 1973) |
|  |  | 36 | Mature (130 cm) | Upper |  |  |  |  |
| *Triakis semifasciata* | Leopard shark | 9-12 | - | - | - | 5 weeks | Spring | (Reif et al., 1978) |
| *Ginglymostoma cirratum* | Nurse shark | 28 | - | - | - | 3 months | Spring |  |
| *Ginglymostoma cirratum* | Nurse shark | 9-21 (summer) | 79.8-136.3 cm 8-14.8 kg | Upper + lower combined | Marked teeth | 3 years | 3 years | (Luer et al., 1990) |
|  |  | 23-40 (winter) |  |  |  |  |  |  |
|  |  | 51-70 (“cold winter”) |  |  |  |  |  |  |
| *Triakis semifasciata* | Leopard shark | 40-55 | - | - | Tooth isotopic composition after diet switch | 3.4 years | 3.4 years | (Zeichner et al., 2017) |


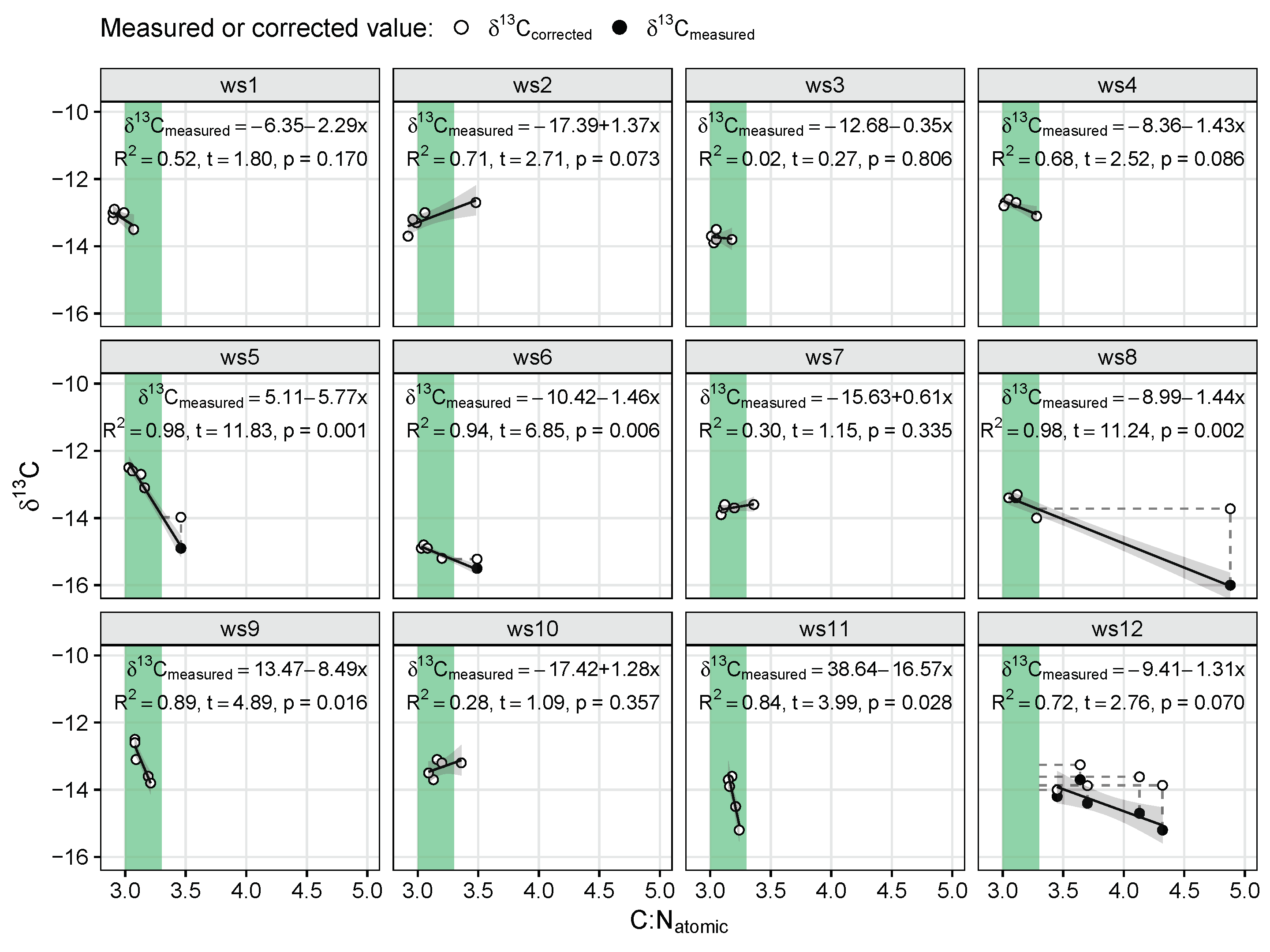
**Figure S1.** Relationships between δ^13^C and atomic C:N ratios (C:N_atomic_) and correction procedures applied to samples of tooth collagen from 12 white sharks (shark ID numbers indicated in panel labels). The green shaded region shows the recommended range of C:N_atomic_ (3.0–3.3) for collagen protein quality control from Guiry and Szpak (2020). For samples with C:N_atomic_ > 3.3 and a significant negative relationship, δ^13^C was corrected (based on a C:N_atomic_ = 3.3) using a scaled offset equation following Shipley et al. (2021). Points and dotted lines show the shifts in δ^13^C values before (δ^13^C_measured_, black dots) and after (δ^13^C_corrected_, white dots) the correction was applied. Equations and results of linear models of the relationship between δ^13^C_measured_ and C:N_atomic_ are listed at the top of each panel. Values for ws12 were corrected due to a high R^2^ which was deemed to be ecologically meaningful despite a lack of statistical significance (p > 0.05), likely resulting from low sample size.


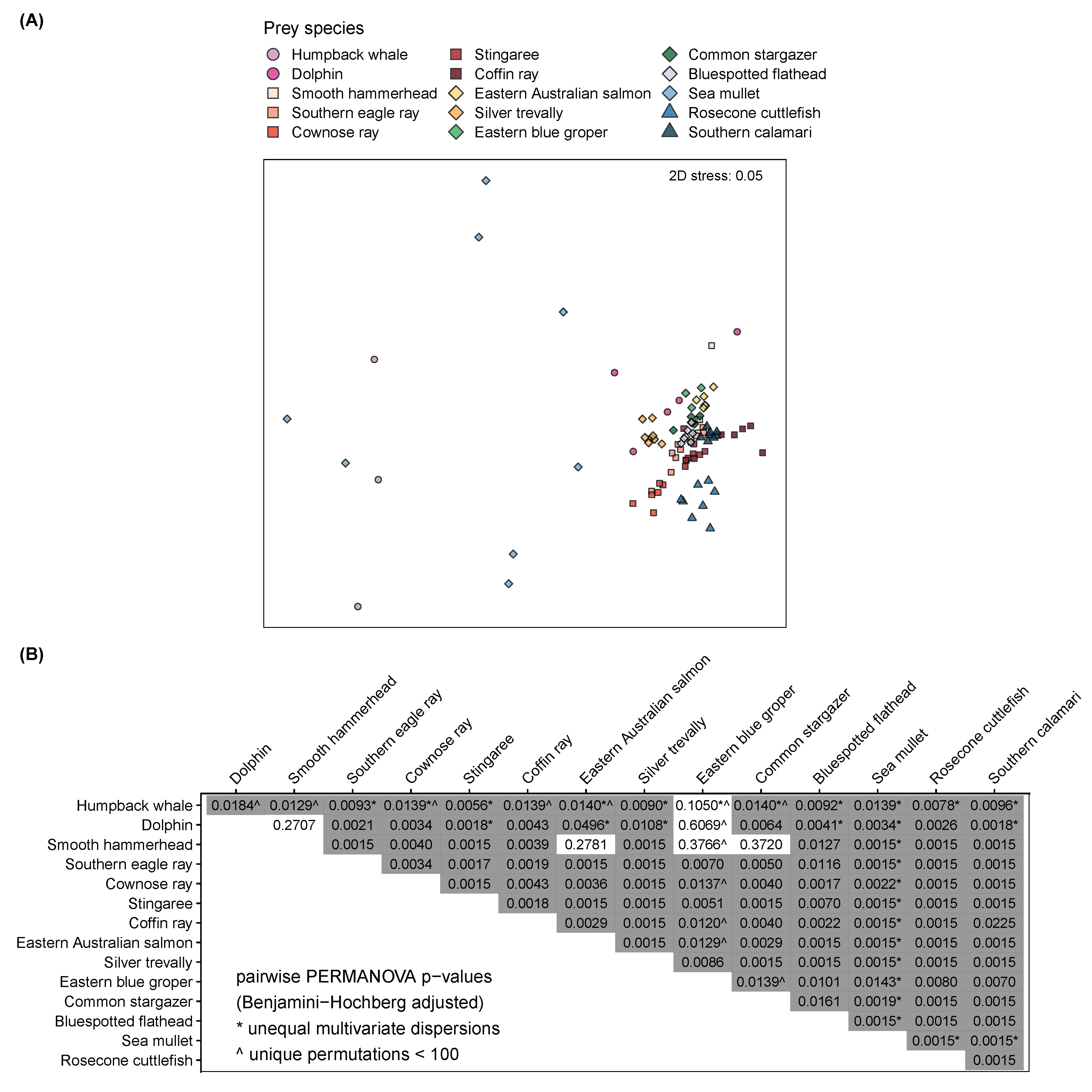


**Figure S2.** (A) Non-metric multidimensional scaling plot based on normalised Euclidean distances of isotopic signatures (δ^13^C, δ^15^N, δ^34^S) for each prey species. Note species of dolphins (*Tursiops truncatus*, n = 3, and *Delphinus delphis*, n = 2) and stingarees (*Urolophus viridis*, n = 3, and *Urolophus paucimaculaus*, n = 7) were pooled prior to analysis into single groups due to low sample sizes and similarity in signatures among individual species. Species are colour coded, and shapes distinguish different functional groups (circles = mammals, squares = elasmobranchs, diamonds = teleosts, triangles = cephalopods). (B) Matrix of p-values from pairwise species PERMANOVA comparisons based on normalised Euclidean distances of isotopic signatures. P-values have been adjusted for multiple comparisons (p.adjust function, stats package; R Core Team, 2021) according to Benjamini and Hochberg (1995), with significant differences are inferred at p < 0.05 (grey tiles). Comparisons where the assumption of equal multivariate dispersions was not met are indicated with “*”, which is mostly for sea mullet (*Mugil cephalus*) and humpback whale (*Megaptera* *novaeangliae*) groups. Despite this, isotopic signatures of these species were obviously different to all other prey. Small sample sizes in humpback whales (n = 3) and Eastern blue groper (*Achoerodus viridis*, n = 3) resulted in some comparisons with a low numbers of unique permutations (< 100 unique pseudo-F values, which are indicated with "^"; Anderson et al., 2008). Nonetheless, for all pairwise comparisons (excluding humpback whales vs eastern blue groper), the minimum possible p-value (1/unique permutations; Anderson et al., 2008) was < α (0.05), meaning the null could be rejected. For humpback whales vs Eastern blue groper, the null could not be rejected given a low number of unique permutations (minimum p-value = 0.1).


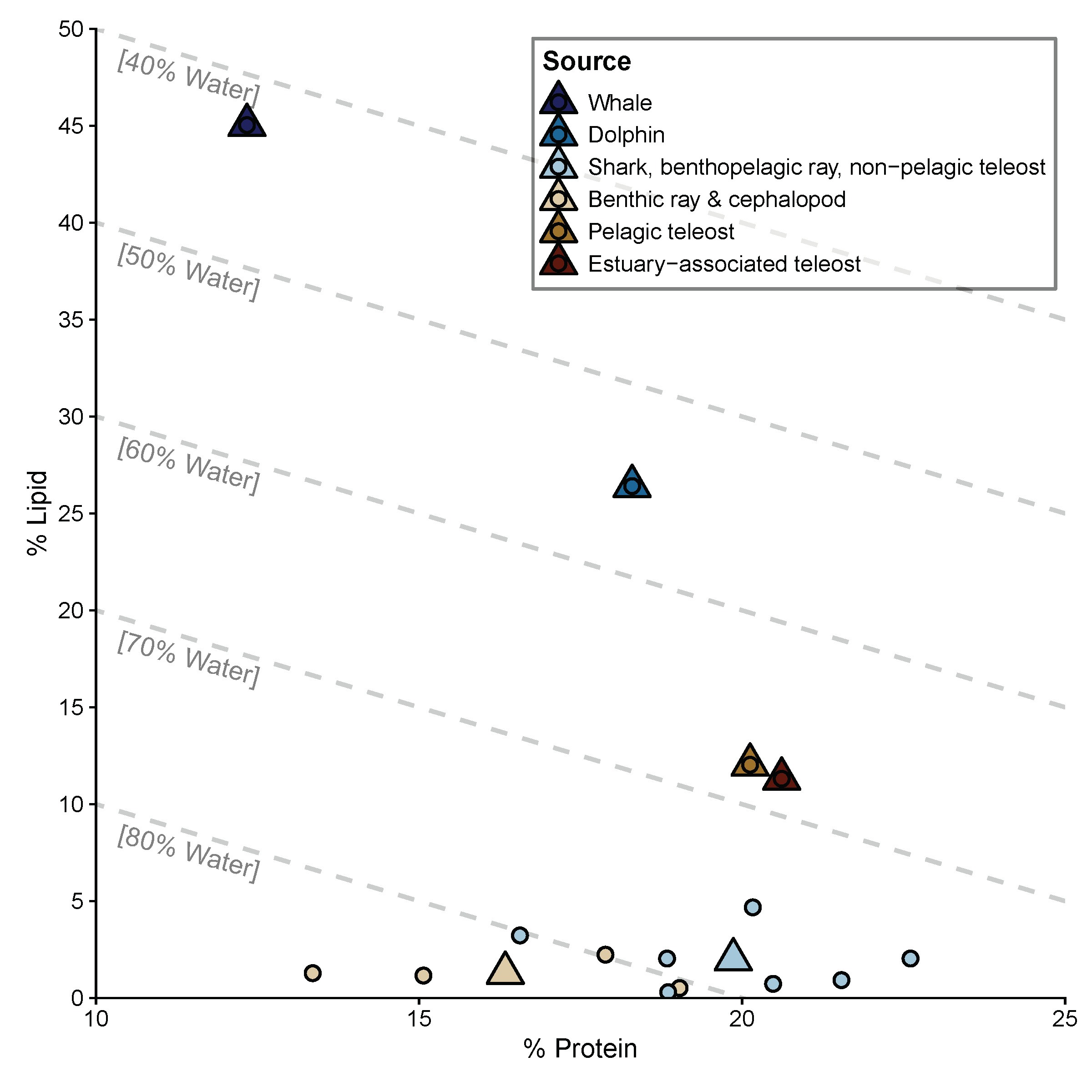


**Figure S3.** Proportions-based nutritional geometry framework model indicating the wet mass % of protein, lipid and water for prey of white sharks in eastern Australia, extracted from the literature. Nutritional compositions of individual prey species (circles) and mean values for source groupings (triangles) are shown. Note that species within source groupings used in the mixing model generally cluster in nutrient space. For instance, benthic rays and cephalopods have both low % protein and lipid, while sharks, benthopelagic rays and non-pelagic teleosts have high % protein and but low % lipid.


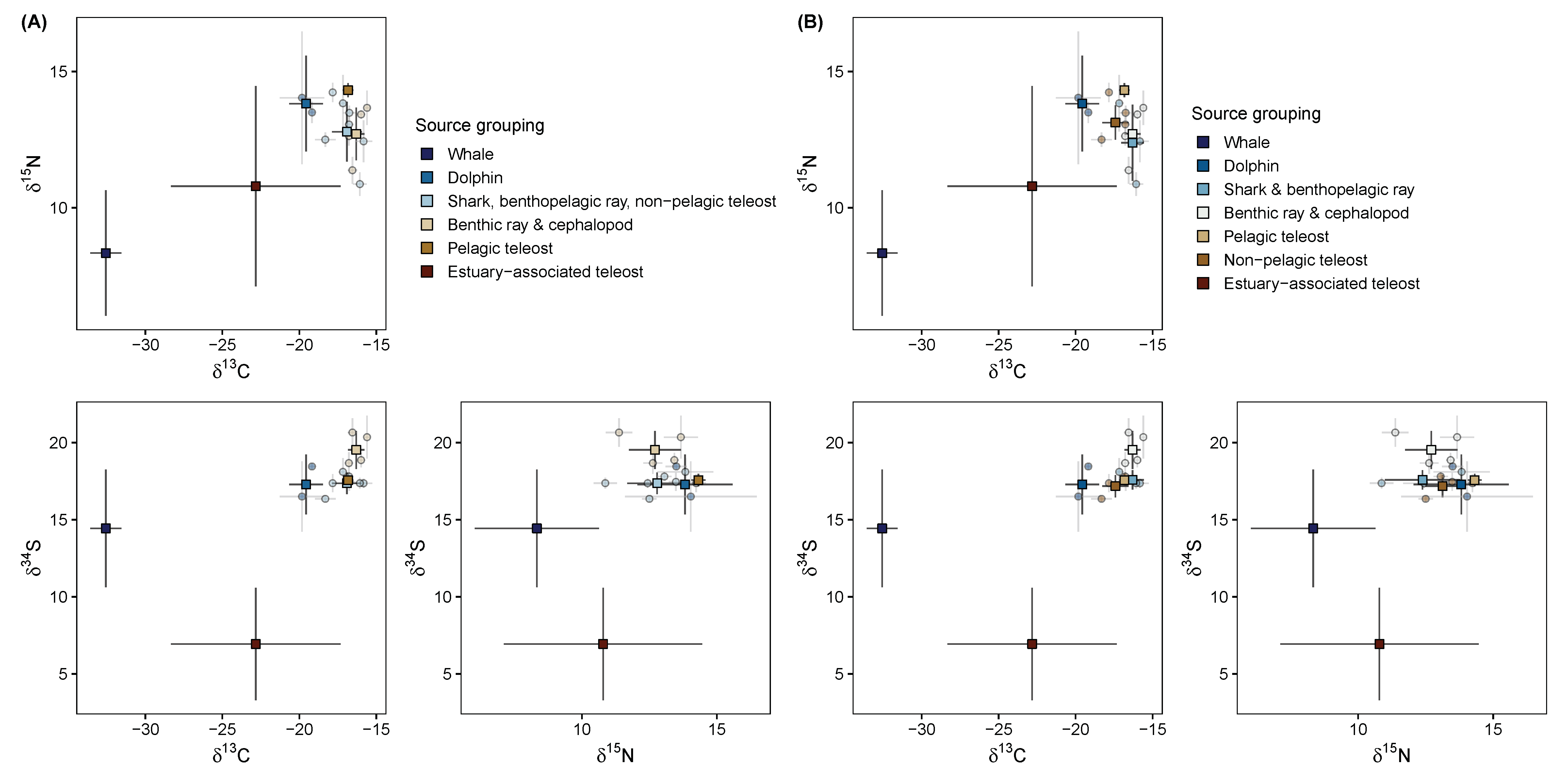
**Figure S4.** (A) Mean ± SD isotopic signatures of individual prey species (circles) and source groupings (squares) used in mixing models, shown on all 3 isotope axis combinations. Whales and estuary-associated teleosts were separated from other species on all axes. Benthic rays and cephalopods were mostly distinguished from other species by δ^34^S. Dolphins were separated from other species by δ^13^C. Pelagic teleosts were distinguished from sharks, benthopelagic rays and non-pelagic teleosts mostly on δ^15^N. Note that high overlap between sharks and benthopelagic rays and non-pelagic teleosts on all isotope axes (B) resulted in these prey being pooled for the mixing model to mitigate impacts on model performance due to difficulties in distinguishing these groups. While this group therefore contained taxonomically variable prey, sharks, benthopelagic rays and non-pelagic teleosts had similar nutritional compositions (Fig. S3), maintaining interpretability for nutritional modelling.


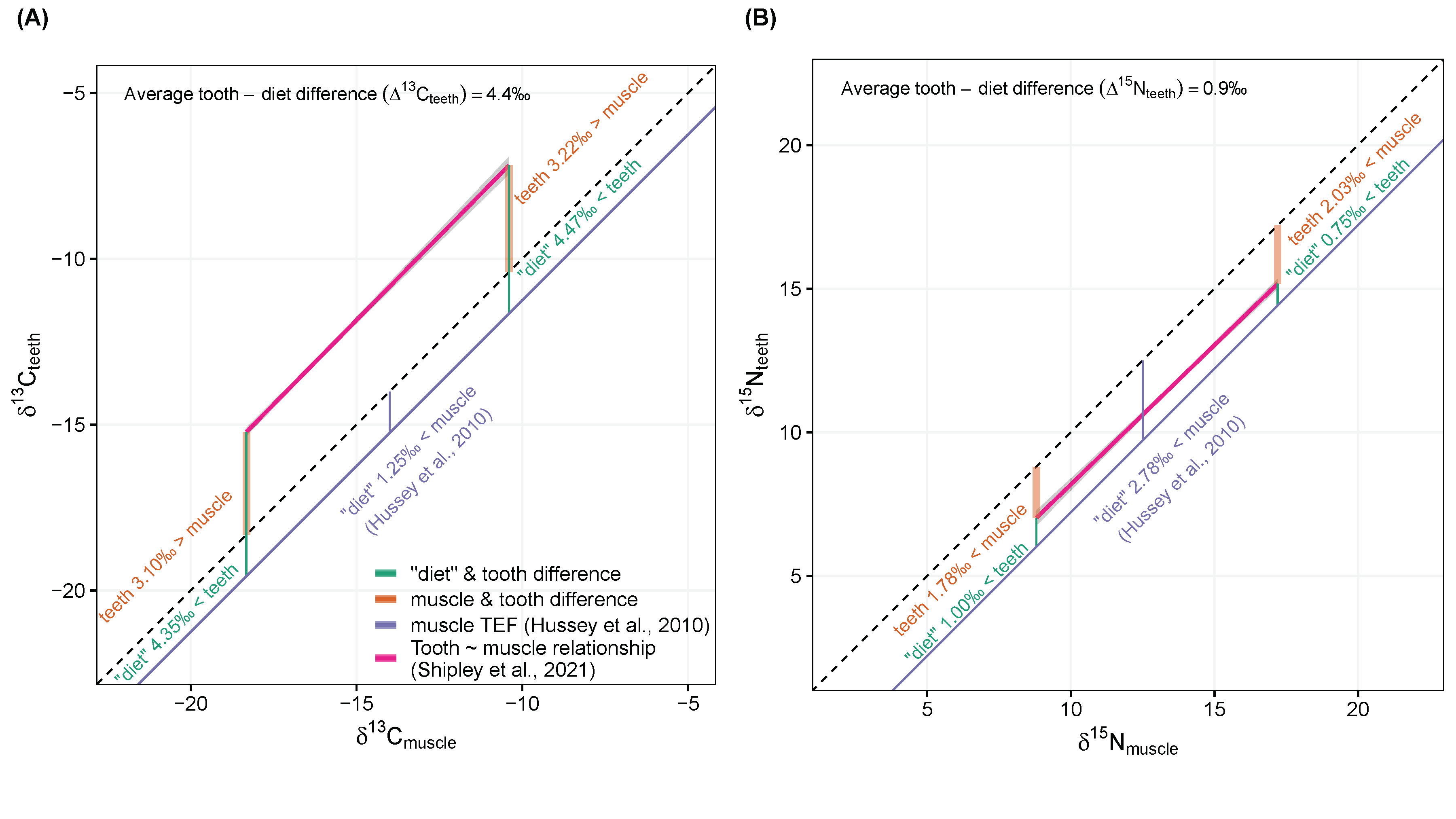


**Figure S5.** Schematic illustration of the indirect method for calculating the TEF_C_ scenario for (A) carbon and (B) nitrogen based on muscle trophic enrichment factors (TEF, Δ^13^C and Δ^15^N) for large sharks fed a bulk (non-lipid extracted), mixed diet from Hussey et al. (2010), and the offset relationships between muscle and tooth isotopic signatures from Shipley et al. (2021). The tooth ~ muscle relationship (pink line) was plotted using the raw data provided in the supplementary material of Shipley et al. (2021) and is the same as presented in that article. The dotted line is the 1:1 relationship, where tooth and muscle signatures would be equal and thus trophic enrichment (Δ^13^C and Δ^15^N) would also be assumed equal. The tooth ~ muscle relationship falls above and below the 1:1 line for (A) δ^13^C and (B) δ^15^N, respectively, suggesting a larger Δ^13^C and smaller Δ^15^N in teeth than muscle. The muscle TEF from Hussey et al. (2010; purple line) is indicated by subtraction from the 1:1 line and shows the value of the “diet”, relative to both muscle (1:1 line) and teeth (pink line). The Δ^13^C and Δ^15^N for the TEF_C_ scenario were computed by adding (δ^13^C) or subtracting (δ^15^N) the value of the tooth ~ muscle offsets (orange lines and text) from the muscle TEF. Maximum and minimum values for the TEF_C_ Δ^13^C and Δ^15^N over the range of predicted values are indicated by green vertical lines and text. The average over 1000 evenly spaced predicted values for tooth ~ muscle relationship was used as the estimate for TEF_C_ for both carbon and nitrogen (Δ^13^C_teeth_ and Δ^15^N_teeth_), which are listed at the top of each plot.

.


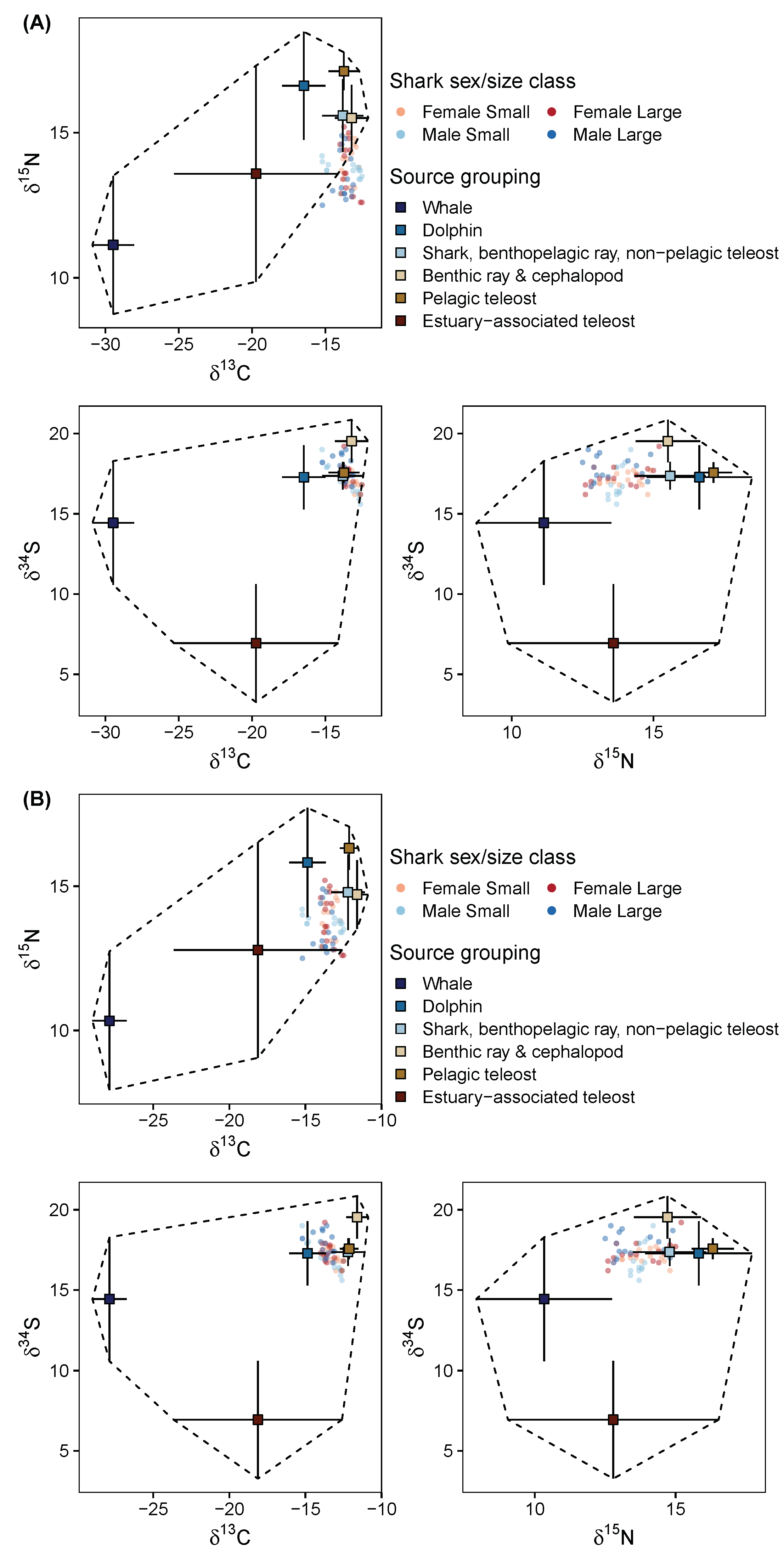


**Figure S6.** Mixing spaces under trophic enrichment scenarios (A) TEF_A_ and (B) TEF_B_ with isotopic signatures of shark tooth samples (circles) and mean ± SD signatures of source groupings (squares) on all 3 isotopic axis combinations (δ^13^C, δ^15^N and δ^34^S). Source signatures are adjusted for trophic enrichment under each scenario, with errors displayed as the combined source + trophic enrichment SD following the formula from Stock and Semmens (2016).


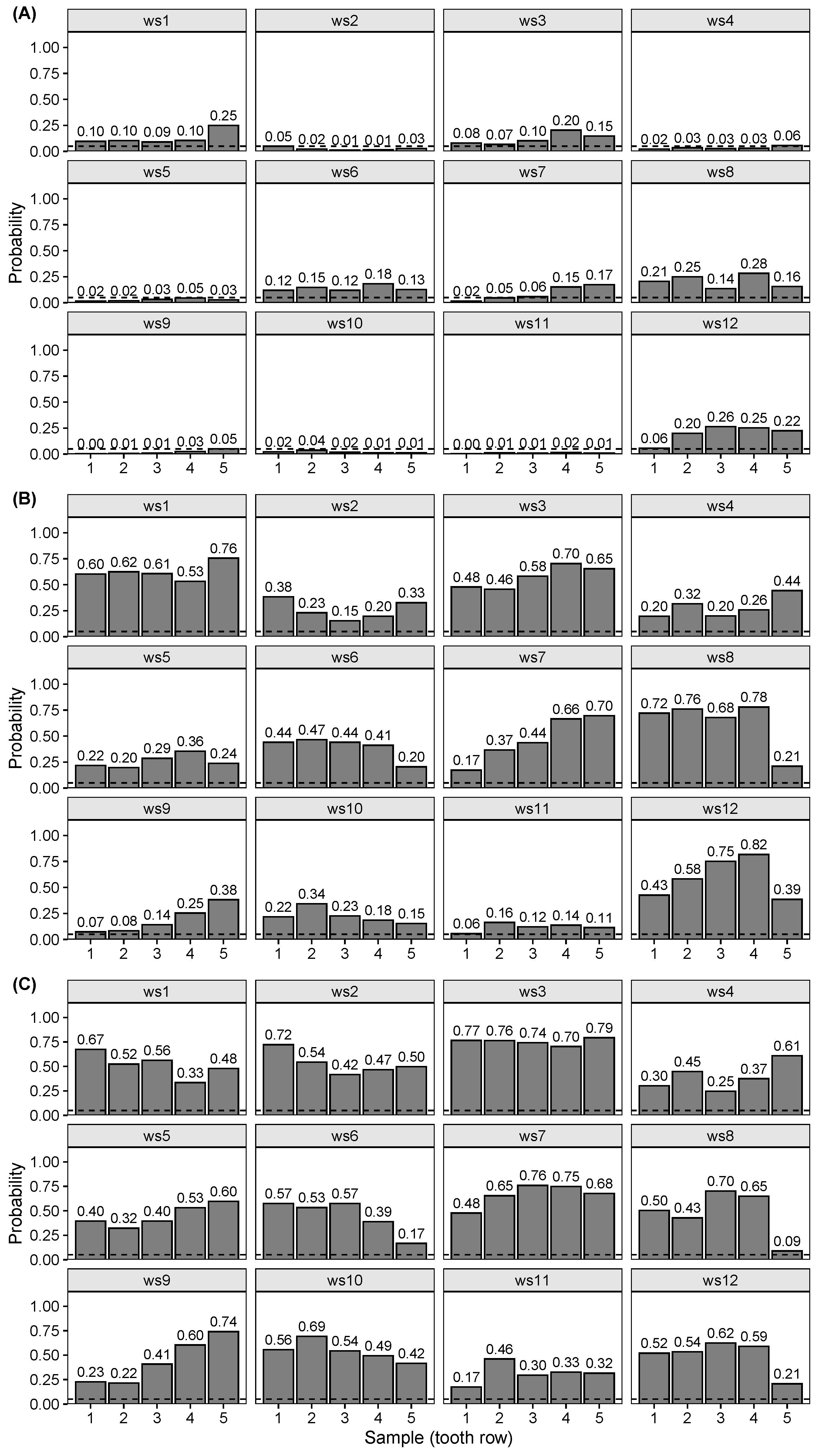


**Figure S7.** Probabilities of consumer (tooth) isotopic signatures falling within simulated mixing polyhedrons under (A) TEF_A_, (B) TEF_B_ and (C) TEF_C_ scenarios. Individual sharks are separated into separate panels, with the probabilities for individual tooth samples (ordered by tooth row) indicated by columns and the corresponding values above. Samples with probabilities >0.05 (horizontal dashed line) fall within the 95% mixing region and were retained in mixing model analyses (Smith et al., 2013).

**Table S2.** Proximate compositions (wet mass percentages of water, protein and lipid) obtained from the literature for prey species consumed by white sharks in eastern Australia and used to model nutritional intakes from mixing model outputs. Where multiple species were grouped into a source, a mean source proximate composition is used. Raw values have been scaled to sum to 100% for plotting with proportions-based nutritional geometry framework models.

| **Source** | **Prey Species** | **Common Name** | **Taxon Used for Composition** | **% Water** | **% Protein** | **% Lipid** | **Reference** |
| --- | --- | --- | --- | --- | --- | --- | --- |
| Whale | *Megaptera novaeangliae* | Humpback whale | *Balaenoptera physalus* | 42.6 | 12.3 | 45.0 | (Lockyer et al., 1985) |
| Dolphin | *Tursiops aduncus* | Bottlenose dolphin | *Tursiops truncatus^A^* | 55.3 | 18.3 | 26.4 | (Dunkin et al., 2005; Hao et al., 2008; Mallette et al., 2016) |
|  | *Delphinus delphis* | Common dolphin | *Tursiops truncatus^A^* | 55.3 | 18.3 | 26.4 |  |
|  |  |  | Source mean | 55.3 | 18.3 | 26.4 |  |
| Shark, benthopelagic ray, non-pelagic teleost | *Sphyrna zygaena* | Smooth hammerhead | *Sphyrna lewini* | 75.4 | 22.6 | 2.0 | (Lowe, 2002) |
|  | *Myliobatis tenuicaudatus* | Southern eagle ray | *Myliobatis peruvianus* | 77.5 | 21.5 | 0.9 | (Sidwell, 1981) |
|  | *Rhinoptera neglecta* | Cownose ray | *Rhinoptera* sp.^B^ | 78.8 | 20.5 | 0.7 | (Sidwell, 1981; Licciardello & Ravesi, 1988) |
|  | *Pseudocaranx georgianus* | Silver Trevally | *Pseudocaranx georgianus* | 75.2 | 20.2 | 4.7 | (Vlieg, 1988) |
|  | *Achoerodus viridis* | Eastern Blue Groper | Labridae | 79.1 | 18.8 | 2.0 | (Sidwell, 1981) |
|  | *Kathetostoma laeve* | Common stargazer | *Kathetostoma giganteum* | 80.2 | 16.6 | 3.2 | (Vlieg, 1988) |
|  | *Platycephalus caeruleopunctatus* | Bluespotted flathead | *Platycephalus indicus* | 80.8 | 18.9 | 0.3 | (Bogard et al., 2015) |
|  |  |  | Source mean | 78.1 | 19.9 | 2.0 |  |
| Benthic ray & cephalopod | *Urolophus* sp. | Stingaree | *Dasyatis* sp.*^C^* | 80.5 | 19.0 | 0.5 | (Sidwell, 1981) |
|  | *Hypnos monopterigius* | Coffin ray | Torpedinidae^D^ | 85.4 | 13.4 | 1.3 | (Sidwell, 1981; Eder & Lewis, 2005) |
|  | *Sepia rozella* | Rosecone cuttlefish | *Sepia sp*.^E^ | 83.8 | 15.1 | 1.2 | (Battam et al., 2010; Spitz et al., 2010) |
|  | *Sepioteuthis australis* | Southern calamari | *Sepioteuthis australis* | 79.9 | 17.9 | 2.2 | (Vlieg, 1988) |
|  |  |  | Source mean | 82.4 | 16.3 | 1.3 |  |
| Pelagic teleost | *Arripis trutta* | Eastern Australian Salmon | *Arripis trutta* | 67.8 | 20.1 | 12.0 | (Vlieg, 1988) |
| Estuary-associated teleost | *Mugil cephalus* | Sea mullet | *Mugil cephalus* | 68.1 | 20.6 | 11.3 | (Vlieg, 1988) |
| ^A^Whole body composition estimated using the proximate composition of blubber and muscle (Dunkin et al., 2005; Hao et al., 2008), weighted by the contribution of each tissue to total body mass (Mallette et al., 2016); ^B^Average for *Rhinoptera jayakari, R. bonasus*; ^C^Average for *Dasyatis pastinaca, D. kuhlii, D. uarnak, D. akajei*; ^D^Average for *Torpedo torpedo*, *Discopyge tschudii*; ^E^Average for *Sepia apama, S. officinalis*. | | | | | | | |

**Table S3.** Small-sample Akaike information criterion (AIC_c_) model comparisons for candidate beta generalised linear models predicting isotopic niche s- and o-indices of white sharks by size class (small, large) and sex (male, female).

| Model | df | AIC_c_ | ΔAIC_c_ |
| --- | --- | --- | --- |
| **s-index ~ size class** | **3** | **-20.9** | **0.0** |
| s-index ~ 1 | 2 | -20.0 | 0.9 |
| s-index ~ sex + size class | 4 | -16.9 | 4.0 |
| s-index ~ sex | 3 | -16.5 | 4.3 |
| **o-index ~ 1** | **2** | **-181.3** | **0.0** |
| o-index ~ sex | 3 | -178.5 | 2.8 |
| o-index ~ size class | 3 | -178.5 | 2.8 |
| o-index ~ sex + size class | 4 | -175.6 | 5.7 |

**Table S4.** Small-sample Akaike information criterion (AIC_c_) model comparisons for candidate beta generalised linear models predicting similarities between individuals and the population in prey proportions (c-index_prey_, p-space) and nutrient intakes (c-index_nutrients_, N-space) by shark size (small, large) and sex (male, female). Model comparisons were conducted separately under each trophic enrichment factor (TEF) scenario.

| Niche space | TEF scenario | Model | df | AIC_c_ | ΔAIC_c_ |
| --- | --- | --- | --- | --- | --- |
| p-space  (prey proportions) | TEF_C_ | **c-index_prey_ ~ 1** | **2** | **-21.5** | **0.0** |
|  |  | c-index_prey_ ~ size class | 3 | -20.9 | 0.6 |
|  |  | c-index_prey_ ~ sex | 3 | -17.9 | 3.6 |
|  |  | c-index_prey_ ~ sex + size class | 4 | -16.3 | 5.2 |
|  | TEF_B_ | **c-index_prey_ ~ 1** | **2** | **-31.7** | **0.0** |
|  |  | c-index_prey_ ~ sex | 3 | -29.3 | 2.5 |
|  |  | c-index_prey_ ~ size class | 3 | -28.4 | 3.3 |
|  |  | c-index_prey_ ~ sex + size class | 4 | -25.0 | 6.8 |
| N-space  (nutrient intakes) | TEF_C_ | **c-index_nutrients_ ~ size class** | **3** | **-102.6** | **0.0** |
|  |  | c-index_nutrients_ ~ 1 | 2 | -100.8 | 1.8 |
|  |  | c-index_nutrients_ ~ sex + size class | 4 | -98.0 | 4.6 |
|  |  | c-index_nutrients_ ~ sex | 3 | -97.3 | 5.3 |
|  | TEF_B_ | **c-index_nutrients_ ~ 1** | **2** | **-101.0** | **0.0** |
|  |  | c-index_nutrients_ ~ size class | 3 | -100.3 | 0.6 |
|  |  | c-index_nutrients_ ~ sex | 3 | -97.4 | 3.5 |
|  |  | c-index_nutrients_ ~ sex + size class | 4 | -95.7 | 5.3 |

**
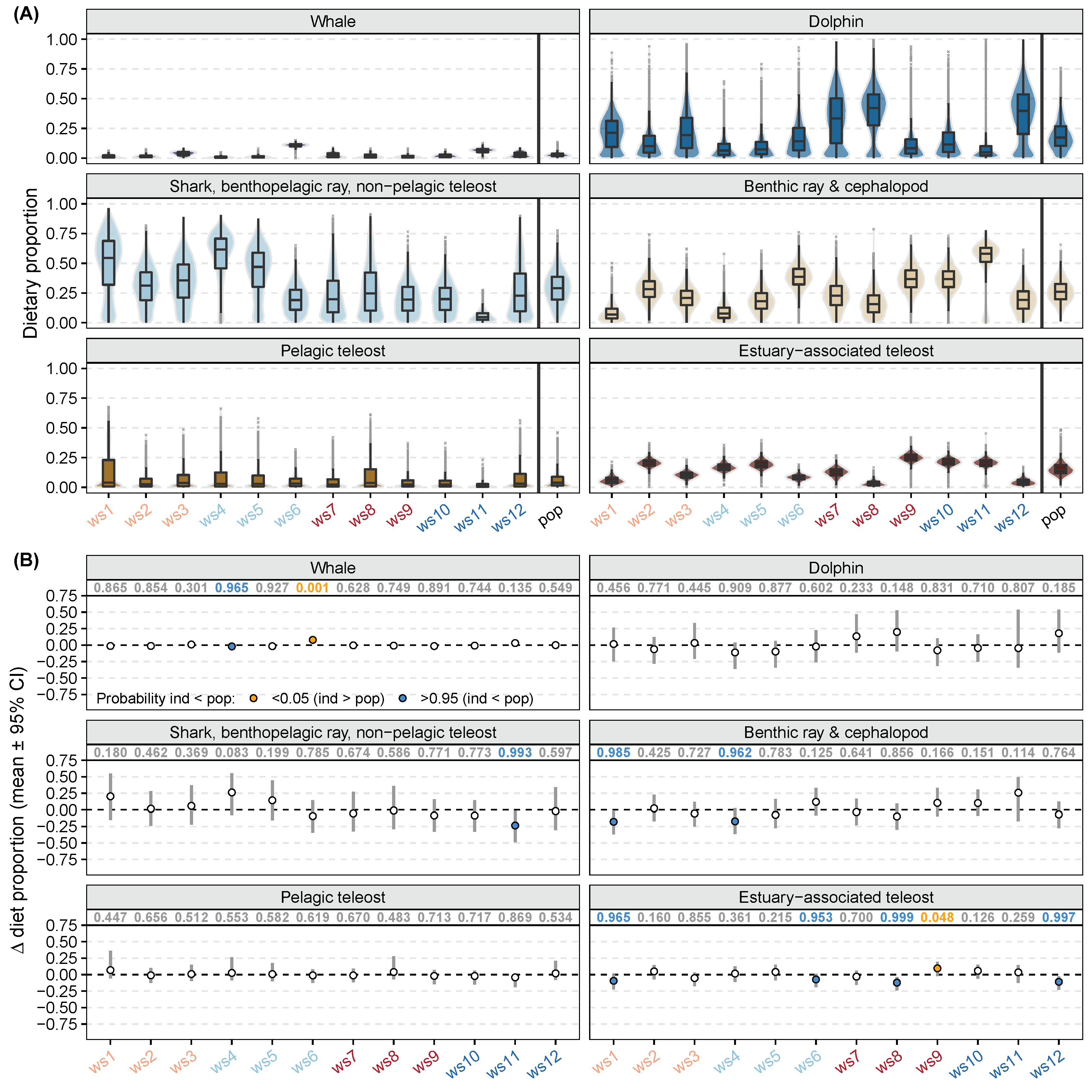
Figure S8.** (A) Violin plots of posterior distributions for the diet proportions of individual white sharks (ind, ws1–ws12) and the overall population (pop) under the TEF_B_ scenario. (B) Mean and 95% credible intervals (CI) of differences between estimated diet proportions of individuals and the population. The probabilities that ind < pop are indicated along the top of each plot for each prey source. Differences were inferred for probabilities > 0.95 (ind < pop) or < 0.05 (ind > pop). Shark ID numbers and colour coding by sex and size class correspond to those used on other figures.


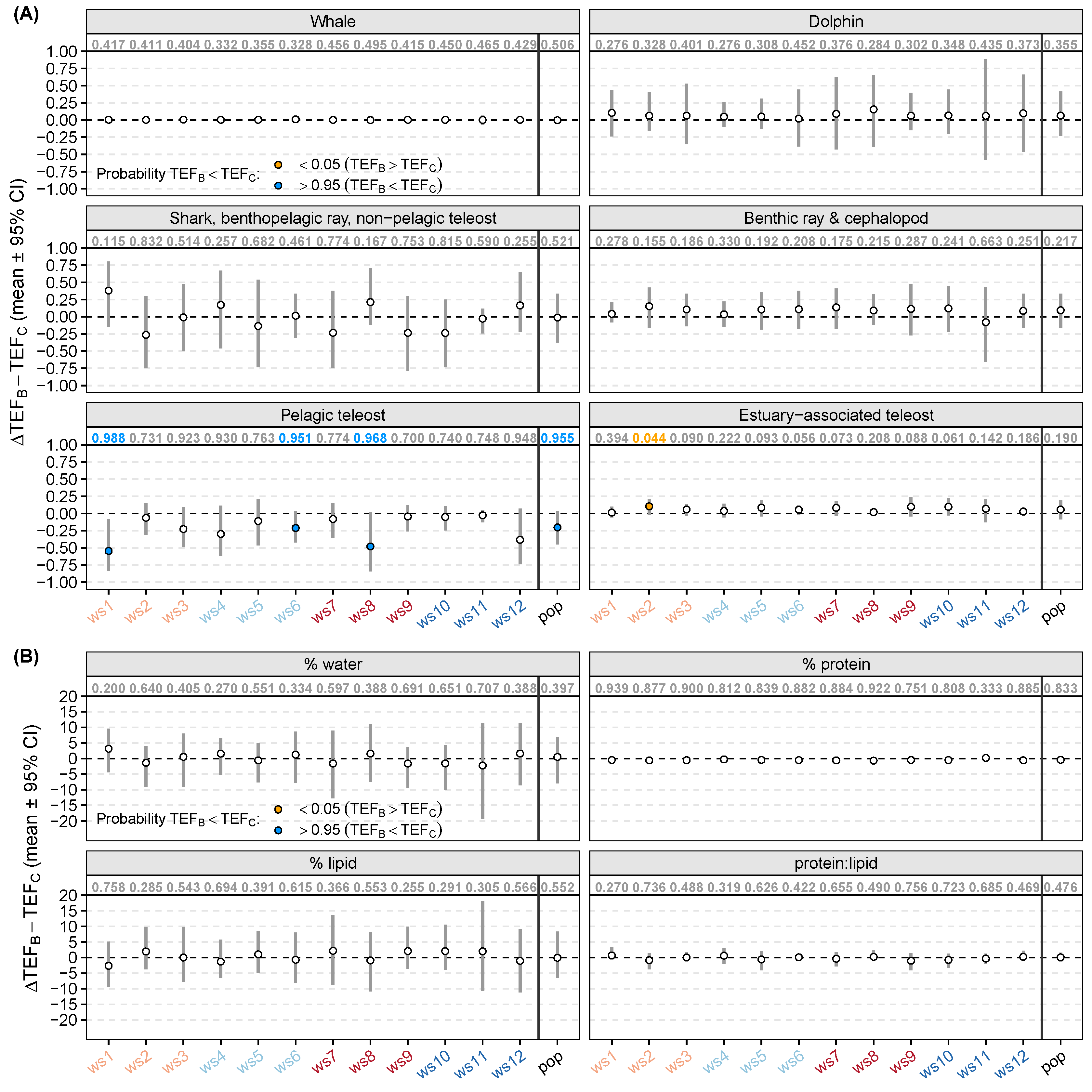
**Figure S9.** Sensitivity analyses showing the mean ± 95% credible intervals (CI) of the differences between outputs of mixing models based on TEF_B_ and TEF_C_ (ΔTEF_B_ – TEF_C_) with respect to estimates for (A) prey proportions, and (B) nutrient intakes for individual white sharks (ws1–ws12) and the overall population (pop). The probabilities that TEF_B_ < TEF_C_ are indicated along the top of each plot for each prey source and nutritional component. Differences were inferred for probabilities > 0.95 (TEF_B_ < TEF_C_) or < 0.05 (TEF_B_ > TEF_C_).

**
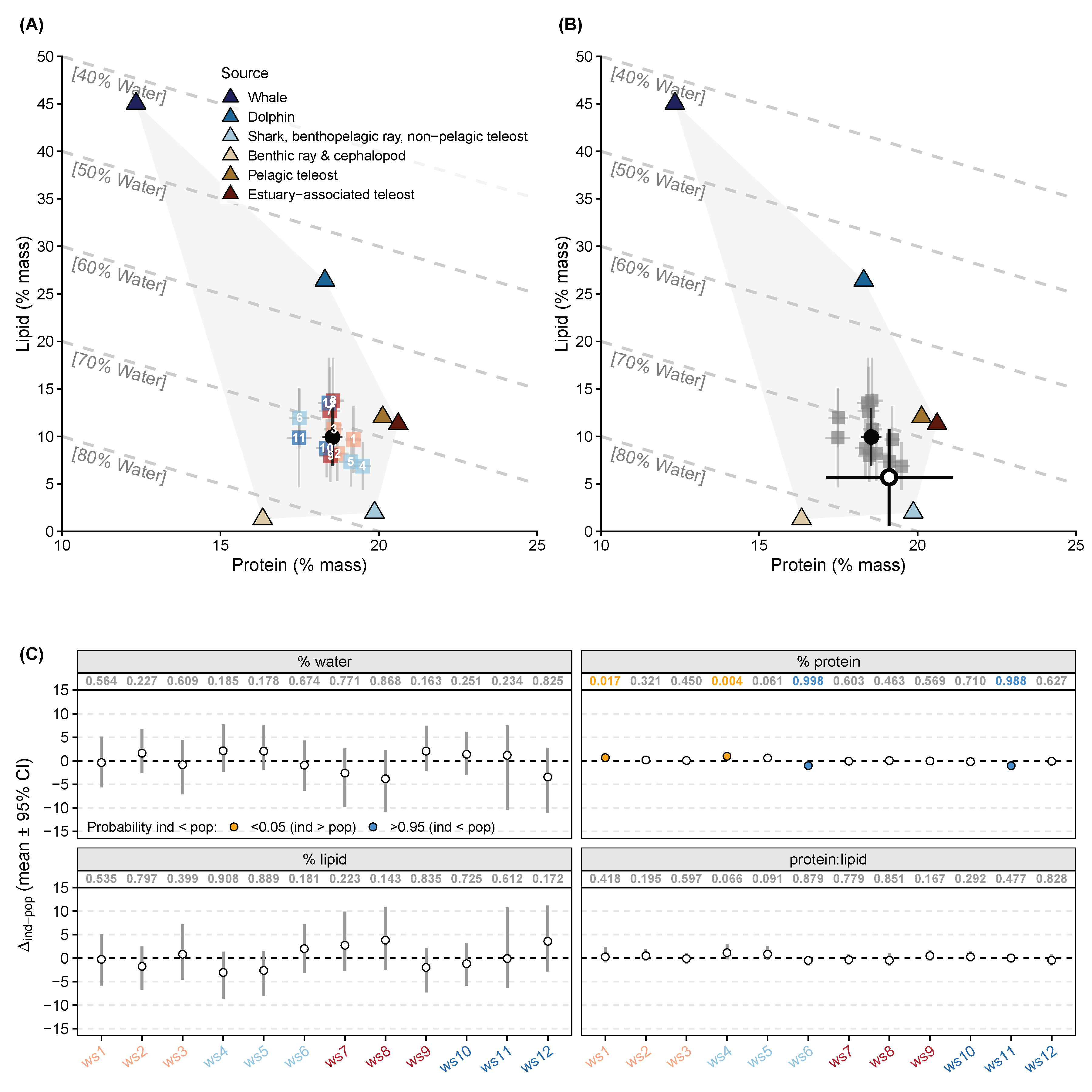
Figure S10.** (A) Proportions-based NGF model of the wet mass % of protein, lipid and water in prey sources (triangles) and diets (posterior mean ± SD) of individual white sharks (squares) and the overall population (black circle) estimated under the TEF_B_ scenario. (B) Mean ± SD nutrient intake for juvenile white sharks based on stomach contents (white circle, n = 40; Grainger et al., 2020) overlayed on mixing model estimates (grey squares = individuals, black circle = population) for comparison. (C) Mean ± 95% credible intervals (CI) of differences between nutrient intakes of each white shark and the overall population (Δ_ind-pop_). The probabilities that ind < pop are displayed along the top of each plot for each nutritional variable. Differences were inferred for probabilities > 0.95 (ind < pop) or < 0.05 (ind > pop). Individual sharks are colour coded for sex and size and labelled with IDs as in other figures.


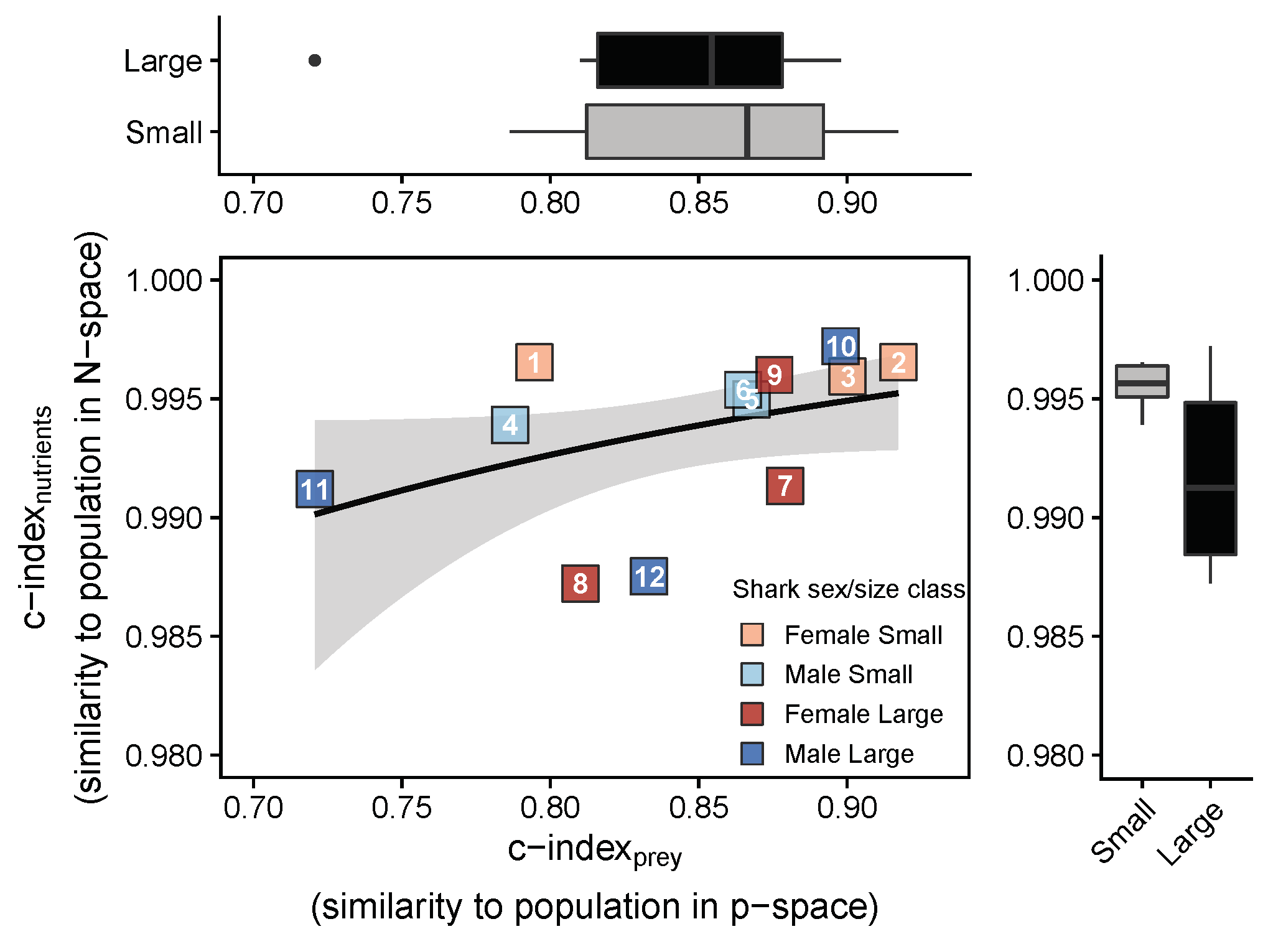


**Figure S11.** Posterior mean cosine similarities between individual white sharks and the overall population based on modelled prey proportions (c-index_prey_, p-space) and nutrient intakes (c-index_nutrients_, N-space) under the TEF_B_ scenario. Marginal boxplots compare variation in c-index_prey_ (top) and c-index_nutrients_ (right) among small (~1.50 m PCL, n = 6) and large (~2.25 m PCL, n = 6) size classes. The predicted relationship (shading = 95% confidence intervals) between the c-index in p- and N-space was not significant (beta GLM, p = 0.069) but is shown to illustrate the deviation of some individuals (e.g. ws1, ws4, ws11) from the expected positive relationship.

**References**

Anderson, M.J., Gorley, R.N., & Clarke, K.R. (2008). *PERMANOVA+ for PRIMER: Guide to Software and Statistical Methods.* PRIMER-E Ltd.

Applegate, S.P. (1967). A survey of shark hard parts. In P.W. Gilbert, R.F. Mathewson & D.P. Rall (eds.) *Sharks, skates and rays* (pp. John Hopkins Press.

Battam, H., Richardson, M., Watson, A.W.T., & Buttemer, W.A. (2010). Chemical composition and tissue energy density of the cuttlefish (*Sepia apama*) and its assimilation efficiency by *Diomedea* albatrosses. *Journal of Comparative Physiology B: Biochemical, Systemic and Environmental Physiology,* *180(8)***,** 1247-1255. <https://doi.org/10.1007/s00360-010-0497-3>.

Benjamini, Y., & Hochberg, Y. (1995). Controlling the False Discovery Rate: a Practical and Powerful Approach to Multiple Testing. *Journal of the Royal Statistical Society: Series B (Methodological),* *57(1)***,** 289-300. <https://doi.org/10.1111/j.2517-6161.1995.tb02031.x>.

Bogard, J.R., Thilsted, S.H., Marks, G.C., Wahab, M.A., Hossain, M.A.R., Jakobsen, J., & Stangoulis, J. (2015). Nutrient composition of important fish species in Bangladesh and potential contribution to recommended nutrient intakes. *Journal of Food Composition and Analysis,* *42***,** 120-133. <https://doi.org/10.1016/j.jfca.2015.03.002>.

Boyne, P.J. (1970). Study of the chronologic development and eruption of teeth in elasmobranchs. *Journal of Dental Research,* *49(3)***,** 556-560. <https://doi.org/10.1177/00220345700490031501>.

Dunkin, R.C., McLellan, W.A., Blum, J.E., & Pabst, D.A. (2005). The ontogenetic changes in the thermal properties of blubber from Atlantic bottlenose dolphin *Tursiops truncatus*. *Journal of Experimental Biology,* *208(8)***,** 1469-1480. <https://doi.org/10.1242/jeb.01559>.

Eder, E.B., & Lewis, M.N. (2005). Proximate composition and energetic value of demersal and pelagic prey species from the SW Atlantic Ocean. *Marine Ecology Progress Series,* *291***,** 43-52. <https://doi.org/10.3354/meps291043>.

Grainger, R., Peddemors, V.M., Raubenheimer, D., & Machovsky-Capuska, G.E. (2020). Diet Composition and Nutritional Niche Breadth Variability in Juvenile White Sharks (*Carcharodon carcharias*). *Frontiers in Marine Science,* *7***,** 422. <https://doi.org/10.3389/fmars.2020.00422>.

Guiry, E.J., & Szpak, P. (2020). Quality control for modern bone collagen stable carbon and nitrogen isotope measurements. *Methods in Ecology and Evolution,* *11(9)***,** 1049-1060. <https://doi.org/10.1111/2041-210x.13433>.

Hao, S., Li, L., Yang, X., Cen, J., Shi, H., Qi, B., & Chen, S. (2008). Character of the Nutritional Composition in Muscle of Bottlenose Dolphin. *Chinese Journal of Zoology,* *43(1)***,** 140-146.

Hussey, N.E., Brush, J., McCarthy, I.D., & Fisk, A.T. (2010). δ^15^N and δ^13^C diet-tissue discrimination factors for large sharks under semi-controlled conditions. *Comparative Biochemistry and Physiology Part A: Molecular & Integrative Physiology,* *155(4)***,** 445-453. <https://doi.org/10.1016/j.cbpa.2009.09.023>.

Ifft, J.D., & Zinn, D.J. (1948). Tooth succession in the smooth dogfish, *Mustelus canis*. *Biological Bulletin,* *95(1)***,** 100-106. <https://doi.org/10.2307/1538156>.

Licciardello, J.J., & Ravesi, E.M. (1988). Frozen storage characteristics of cownose ray (*Rhinoptera bonasus*). *Journal of Food Quality,* *11(1)***,** 71-76. <https://doi.org/10.1111/j.1745-4557.1988.tb00867.x>.

Lockyer, C.H., McConnell, L.C., & Waters, T.D. (1985). Body condition in terms of anatomical and biochemical assessment of body fat in North Atlantic Fin and Sei whales. *Canadian Journal of Zoology,* *63(10)***,** 2328-2338. <https://doi.org/10.1139/z85-345>.

Lowe, C.G. (2002). Bioenergetics of free-ranging juvenile scalloped hammerhead sharks (*Sphyrna lewini*) in Kane'ohe Bay, O'ahu, HI. *Journal of Experimental Marine Biology and Ecology,* *278(2)***,** 141-156. <https://doi.org/10.1016/s0022-0981(02)00331-3>.

Luer, C.A., Blum, P.C., & Gilbert, P.W. (1990). Rate of Tooth Replacement in the Nurse Shark, *Ginglymostoma cirratum*. *Copeia,* *1990(1)***,** 182-191. <https://doi.org/10.2307/1445834>.

Mallette, S.D., McLellan, W.A., Scharf, F.S., Koopman, H.N., Barco, S.G., Wells, R.S., & Pabst, D.A. (2016). Ontogenetic allometry and body composition of the common bottlenose dolphin (*Tursiops truncatus*) from the US mid-Atlantic. *Marine Mammal Science,* *32(1)***,** 86-121. <https://doi.org/10.1111/mms.12253>.

Markel, V.K., & Laubier, L. (1969). Zum Zahnerzatz bei Elasmobranchiern. *Zoologische Beiträge,* *15(1)***,** 41-44.

Moss, S.A. (1967). Tooth replacement in the lemon shark, *Negaprion brevirostris*. In P.W. Gilbert, R.F. Mathewson & D.P. Rall (eds.) *Sharks, skates and rays* (pp. John Hopkins Press.

R Core Team (2021). R: A language and environment for statistical computing., version 4.1.1, <https://www.R-project.org/>,

Reif, W.E., McGill, D., & Motta, P. (1978). Tooth replacement rates of the sharks *Triakis semifasciata* and *Ginglymostoma cirratum*. *Zoologische Jahrbücher,* *99***,** 151-156.

Shipley, O.N., Henkes, G.A., Gelsleichter, J., Morgan, C., Schneider, E.V., Talwar, B., & Frisk, M.G. (2021). Shark tooth collagen stable isotopes (δ^15^N and δ^13^C) as ecological proxies. *Journal of Animal Ecology,* *90(9)***,** 2188-2201. <https://doi.org/10.1111/1365-2656.13518>.

Sidwell, V.D. (1981). *Chemical and nutritional composition of finfishes, whales, crustaceans, mollusks, and their products.* National Oceanic and Atmospheric Administration.

Smith, J.A., Mazumder, D., Suthers, I.M., & Taylor, M.D. (2013). To fit or not to fit: evaluating stable isotope mixing models using simulated mixing polygons. *Methods in Ecology and Evolution,* *4(7)***,** 612-618. <https://doi.org/10.1111/2041-210x.12048>.

Spitz, J., Mourocq, E., Schoen, V., & Ridoux, V. (2010). Proximate composition and energy content of forage species from the Bay of Biscay: high- or low-quality food? *ICES Journal of Marine Science,* *67(5)***,** 909-915. <https://doi.org/10.1093/icesjms/fsq008>.

Stock, B., & Semmens, B. (2016). MixSIAR GUI user manual, Version 3.1, <https://github.com/brianstock/MixSIAR>, <https://doi.org/10.5281/zenodo.1209993>.

Vlieg, P. (1988). *Proximate composition of New Zealand marine finfish and shellfish.* Biotechnology Division, Deptartment of Scientific and Industrial Research.

Wass, R.C. (1973). Size, growth, and reproduction of the sandbar shark, *Carcharhinus milberti*, in Hawaii. *Pacific Science,* *27(4)*.

Zeichner, S.S., Colman, A.S., Koch, P.L., Polo-Silva, C., Galvan-Magana, F., & Kim, S.L. (2017). Discrimination Factors and Incorporation Rates for Organic Matrix in Shark Teeth Based on a Captive Feeding Study. *Physiological and Biochemical Zoology,* *90(2)***,** 257-272. <https://doi.org/10.1086/689192>.
